# Enhanced Efficiency and Stability of Tin Halide Perovskite Solar Cells Through MOF Integration

**DOI:** 10.1002/smll.202411346

**Published:** 2025-01-26

**Authors:** Yongqi Yin, Xisheng Zhang, Ho Ngoc Nam, Quan Manh Phung, Kuina Yuan, Boyuan Li, Fanyue Kong, Azhar Alowasheeira, Baoning Wang, Lin Li, Yusuke Yamauchi

**Affiliations:** ^1^ Key Laboratory for Photonic and Electronic Bandgap Materials Ministry of Education School of Physics and Electronic Engineering Harbin Normal University Harbin 150025 China; ^2^ Department of Materials Process Engineering Graduate School of Engineering Nagoya University Furo‐cho, Chikusa‐ku Nagoya 464–8603 Japan; ^3^ Department of Chemistry Graduate School of Science Nagoya University Furo‐cho, Chikusa‐ku Nagoya 464–8602 Japan; ^4^ Institute of Transformative Bio‐Molecules (WPI‐ITbM) Nagoya University Furo‐cho, Chikusa‐ku Nagoya 464–8601 Japan; ^5^ Australian Institute for Bioengineering and Nanotechnology (AIBN) The University of Queensland Brisbane Queensland 4072 Australia; ^6^ Department of Plant & Environmental New Resources and Graduate School of Green‐Bio Science Kyung Hee University 1732 Deogyeong‐daero, Giheung‐gu Yongin‐si Gyeonggi‐do 17104 South Korea

**Keywords:** efficiency, FASnI_3_, MOFs, stability, tin halide perovskite solar cells

## Abstract

Tin halide perovskites are promising candidates for lead‐free perovskite solar cells due to their ideal bandgap and high charge‐carrier mobility. However, poor crystal quality and rapid degradation in ambient conditions severely limit their stability and practical applications. This study demonstrates that incorporating UiO‐66, a zirconium‐based MOF, significantly enhances the performance and stability of tin halide perovskite solar cells (TPSCs). The unique porous structure and abundant carboxylate groups of UiO‐66 improve the crystallinity and film quality of FASnI₃, reduce defect density, and prolong charge carrier lifetimes. Consequently, the power conversion efficiency (PCE) of UiO‐66‐integrated TPSCs increases from 11.43% to 12.64%, and the devices maintain over 90% of their initial PCE after 100 days in a nitrogen glovebox. These findings highlight the potential of UiO‐66 in addressing the efficiency and stability challenges of tin halide perovskites.

## Graphical abstract

Tin halide perovskite solar cells face challenges in stability and efficiency. This study integrates UiO‐66, a zirconium‐based MOF, enhancing crystallinity, reducing defects, and extending carrier lifetimes in FASnI_3_. Power conversion efficiency increased from 11.43% to 12.64%, and stability improved significantly. These findings underscore the potential of MOF integration in advancing lead‐free perovskite photovoltaics.

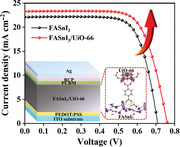

## 1 Introduction

Organic–inorganic hybrid lead halide perovskites have garnered significant attention due to their exceptional power conversion efficiency (PCE), cost‐effectiveness, and lightweight applications, making them a focal point in photovoltaic research.^[^ ^1^, ^2^, ^3^, ^4^, ^5^ ^]^ However, the toxicity and environmental risks associated with lead‐based perovskites restrict their practical applications, driving the search for lead‐free alternatives.^[^ ^6^, ^7^, ^8^, ^9^ ^]^ Among various candidates, tin (Sn) halide perovskites have emerged as promising substitutes due to their suitable bandgap (1.2–1.4 eV), lower exciton binding energy (18 meV), and higher charge‐carrier mobility, which potentially enable high PCE.^[^ ^10^, ^11^ ^]^

Despite these advantages, tin halide perovskite solar cells (TPSCs) face substantial stability challenges that limit their practical applications and long‐term performance. Sn^2^⁺ easily oxidizes to Sn⁴⁺ in the presence of oxygen and moisture, creating tin vacancies that accelerate material degradation. Its reactive 5s electrons make Sn^2^⁺ more prone to oxidation than Pb^2^⁺. Oxidation products like SnO₂, SnI₄, and I₂ accumulate at grain boundaries or surfaces, worsening degradation and reducing the perovskite layer's light absorption and charge transport.^[^ ^12^, ^13^, ^14^ ^]^ Additionally, the p‐type doping caused by Sn^2+^ oxidation leads to high defect densities and increased carrier recombination rates, significantly lowering the open‐circuit voltage (*V*_oc_) and fill factor (FF), ultimately diminishing the device's efficiency and stability.^[^ ^15^, ^16^, ^17^ ^]^ Besides the oxidation issues, TPSCs also suffer from rapid crystallization, poor film quality, and high defect density, all of which limit carrier diffusion length and light‐harvesting efficiency. These challenges further reduce TPSCs' efficiency and stability, constraining their potential as viable lead‐free alternatives.^[^ ^18^, ^19^, ^20^ ^]^

To address these challenges, researchers have explored various methods, including A‐site cation engineering with Cs^+^ to improve crystal quality and using long‐chain organic ammonium ions (e.g., PEA^+^, BA^+^) to protect against moisture and oxygen erosion.^[^ ^21^, ^22^ ^]^ Additionally, antioxidants or protective layers, such as reducing agents or barrier layers like N_2_H_5_Cl, 4‐fluorobenzohydrazide, hydroquinone sulfonic acid, sodium borohydride, and PMMA, have been introduced to slow the oxidation of Sn^2+^.^[^ ^23^, ^24^, ^25^, ^26^, ^27^ ^]^ Lewis acid‐base additives have also shown significant effects in enhancing film quality and improving TPSCs' efficiency and stability. These strategies have collectively increased TPSC efficiency from 9% to 15% over the past five years.^[^ ^28^, ^29^, ^30^, ^31^ ^]^ However, achieving both high efficiency and long‐term stability remains a critical challenge.

In recent years, metal–organic frameworks (MOFs) have attracted significant attention for their potential applications in enhancing perovskite solar cells' performance. MOFs are crystalline materials composed of metal ions coordinated to organic ligands, forming porous structures with diverse chemical functionalities.^[^ ^32^, ^33^, ^34^ ^]^ The unique properties of MOFs, such as large surface area, tunable pore size, and rich chemical coordination sites, make them potential candidates for improving the crystallization process and protecting perovskite films from environmental degradation.^[^ ^35^, ^36^, ^37^ ^]^ Notably, the functional groups on MOFs can interact with perovskite materials at the molecular level, potentially creating strong coordination bonds or hydrogen bonds that improve film integrity and reduce defect density. For instance, MOFs have been successfully integrated into lead‐based perovskite solar cells, demonstrating significant improvements in both efficiency and stability through such molecular interactions.^[^ ^38^, ^39^, ^40^, ^41^ ^]^ However, MOFs are rarely used in TPSCs, possibly due to the sensitivity of Sn^2+^ and the large molecular size of MOFs, which may influence perovskite layer quality.

In this study, we aim to explore how MOF functional groups can interact with the tin‐based perovskite layer, thus enhancing both film stability and device performance. Herein, UiO‐66, a moisture and chemical‐stable zirconium‐carboxylate MOF (Zr_6_O_4_(OH)_4_(BDC)_6_, BDC = 1,4‐benzenedicarboxylate), was used in this work to enhance the performance of TPSCs. The obtained monodisperse UiO‐66 crystals have a well‐defined shape and tunable sizes ranging from 200–300 nm to 30–50 nm using an acetic acid modulation strategy to adapt the thickness of tin‐based perovskites. The unique porous structure and rich chemical coordination sites of UiO‐66 improve the crystallization process and achieve a high‐quality FASnI₃ film. Theoretical investigations reveal that Sn^2^⁺ in FASnI₃ interacts strongly with the carboxylate groups of UiO‐66, making them the preferred binding sites. Additionally, van der Waals interactions between the Zr_6_O_4_(OH)_4_ nodes of UiO‐66 and the FASnI₃ surface further stabilize the film structure. These interactions significantly reduce defect density and enhance crystallinity, as evidenced by the enhanced photoluminescence (PL) intensity and prolonged time‐resolved photoluminescence (TRPL) lifetime. The absorbance of UiO‐66‐integrated tin perovskites is also increased, as shown by the UV−vis spectrum. Consequently, the efficiency of the TPSCs increased from 11.43% to 12.64%. Moreover, the PCE of the control TPSCs decreased to 38% of its initial value within 144 h, whereas the UiO‐66 integrated TPSCs maintained over 80% of their initial PCE for 240 h under ambient conditions. The UiO‐66 integrated TPSCs maintained over 90% of their initial PCE in a nitrogen flow glovebox for over 100 days.

## 2 Results and Discussion

**Figure 1:**
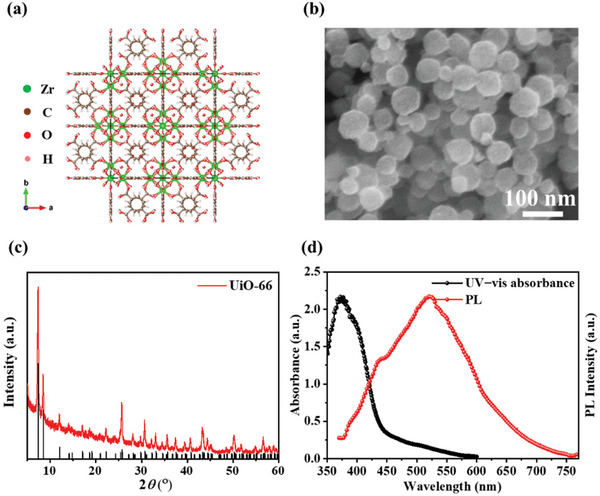
a) The structure of UiO‐66, b) SEM image of UiO‐66, c) XRD pattern of UiO‐66, the bottom black line is simulated pattern, and d) UV−vis absorbance spectrum and PL intensity of UiO‐66.

In this study, the zirconium‐based MOF, UiO‐66, was used to introduce to TPSCs. The structure of the UiO‐66 is illustrated in **Figure** **1**a. UiO‐66, with the formula Zr_6_O_4_(OH)_4_(BDC)_6_, consists of a secondary building unit of Zr_6_O_4_(OH)_4_ cluster and an organic linker of BDC. Details of the synthesis process are provided in the experimental section. Notably, we controlled the size of UiO‐66 by adjusting the synthesis conditions, achieving a reduction in the grain size from 200−300 nm to 30−50 nm, as observed from scanning electron microscope (SEM) images of Figure 1b and Figure S1 (Supporting Information). The smaller size distribution of UiO‐66 is the prerequisite for obtaining a smooth FASnI_3_ perovskite film since the thickness of FASnI_3_ perovskite is ≈200 nm. The X‐ray diffraction (XRD) pattern of UiO‐66 is shown in Figure 1c, where all peaks match well with the simulated peaks from previous reports, confirming the high crystallinity and purity of UiO‐66.^[^ ^38^, ^39^ ^]^ The UV–vis absorption and PL spectra of UiO‐66 are presented in Figure 1d. UiO‐66 exhibits strong absorption in the UV region (350−400 nm) and broad emission in the visible range. These absorption and emission wavelengths are highly suitable for TPSCs, as they help convert harmful UV light into beneficial visible light, thereby enhancing light absorption and electronic conversion.^[^ ^38^, ^39^ ^]^

**Figure 2:**
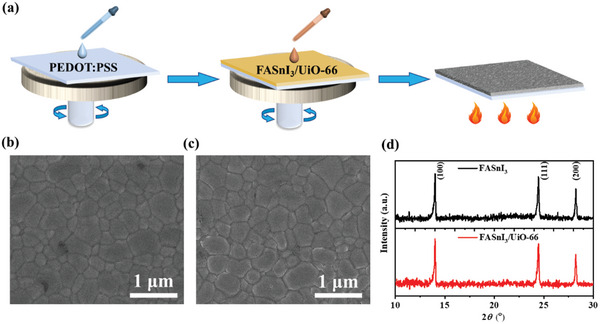
a) Schematic representation of the one‐step spin‐coating method for fabricating FASnI_3_ and FASnI_3_/UiO‐66 perovskite films. The plan‐view SEM images of b) as‐deposited FASnI_3_ and c) FASnI_3_/UiO‐66 perovskite films, respectively. d) XRD patterns of as‐deposited FASnI_3_ and FASnI_3_/UiO‐66 perovskite films.

Subsequently, we introduce the UiO‐66 to tin halide perovskite films by the one‐step spin‐coating method to test the influence of UiO‐66 on the performance of TPSCs. The details can be found in the experimental section, and the schematic representation is shown in **Figure** **2**a. In brief, the hole transport layer PEDOT:PSS is first spin‐coated on the ITO glass, and the perovskite solution with or without UiO‐66 is spin‐coated on the PEDOT:PSS by one‐step spin‐coating method. Finally, the as‐prepared perovskite films are annealed on the hot plate at 100 °C for 10 min to crystallize.

Afterward, the SEM images of FASnI_3_ and FASnI_3_/UiO‐66 perovskite films are shown in Figure 2b,c, respectively. Both films are smooth and without pinholes. The grain size increased slightly from 516 ± 186 nm to 633 ± 235 nm with the incorporation of UiO‐66. Moreover, we examined the XRD patterns of the control and the UiO‐66 integrated perovskite films. As shown in Figure 2d, both films exhibit an orthorhombic lattice structure with characteristic peaks at (100), (111), and (200), without any other peaks, indicating that UiO‐66 does not cause ion‐level doping in the crystal lattice. The full width at half maximum (FWHM) of the (100) peak decreased from 0.12° to 0.1° for FASnI_3_ and FASnI_3_/UiO‐66, respectively, which suggests a higher degree of order and an increase in grain size, which are also consistent with the SEM images.

**Figure 3:**
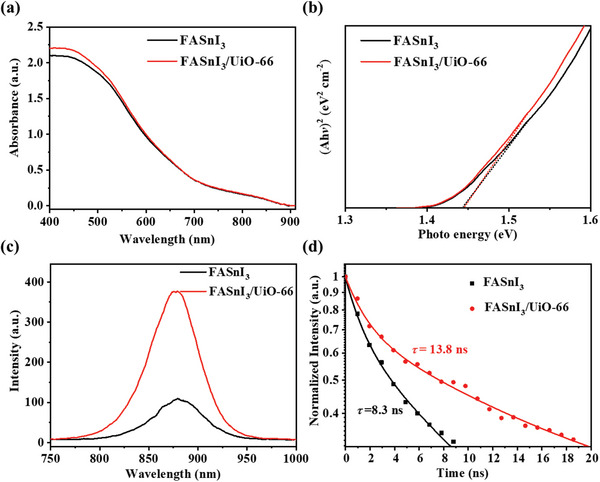
Characterization of FASnI_3_ and FASnI_3_/UiO‐66 perovskite films. a) UV−vis absorbance spectra, b) Tacu plot, c) PL intensity, and d) the TRPL lifetime.

Besides the morphology and structure, it is more important to understand the impact of UiO‐66 on the optical performance of FASnI_3_ since the optical properties of the absorber layer can have a more profound effect on the final characteristics of the TPSCs. **Figure** **3**a shows the UV−vis absorbance spectra of FASnI_3_ and FASnI_3_/UiO‐66 films. The FASnI_3_/UiO‐66 film exhibits higher absorbance in the 400−550 nm range than the FASnI_3_ film. This enhanced absorbance suggests that the incorporation of UiO‐66 improves the light‐harvesting efficiency of the perovskite films by capturing more photons in this wavelength range and converting them into electrons.

Figure 3b presents the Tauc plots of both films, revealing similar bandgap values of ≈1.42 eV for FASnI_3_ and FASnI_3_/UiO‐66 films. This indicates that the addition of UiO‐66 does not alter the intrinsic bandgap of the FASnI_3_ perovskite, ensuring that the fundamental electronic properties are maintained. Further analysis of the PL intensity (Figure 3c) shows that the FASnI_3_/UiO‐66 film exhibits significantly higher PL intensity compared to the FASnI_3_ film, with an increase from ≈100 to ≈380 arbitrary units. In addition, the TRPL lifetime measurements (Figure 3d; Figure S2, Supporting Information) also reveal distinct improvements in PL lifetimes upon incorporating UiO‐66. The PL lifetime of pristine FASnI₃ is 8.3 ns, which increases to 13.8 ns for FASnI₃/UiO‐66. For films using PEDOT:PSS as the hole transport layer, the PL lifetime shows 2.8 ns for FASnI₃ and 2.6 ns for FASnI₃/UiO‐66.

Using Equation (1):

(1) $$L_{D}\approx \frac{2L}{\pi }\sqrt{2\left(\frac{\tau }{\tau_{q}}-1\right)}$$

where *L* is the film thickness, *τ* is the PL lifetime of the perovskite film, and *τ_q_* is the PL lifetime on PEDOT:PSS.^[^ ^42^ ^]^ We calculated the hole diffusion lengths as ≈278 nm for FASnI_3_ and ≈547 nm for FASnI_3_/UiO‐66. This extended diffusion length in FASnI_3_/UiO‐66 benefits charge transport and reduces recombination. PL and TRPL analyses collectively indicate that the incorporation of UiO‐66 significantly enhances the optical properties of FASnI₃ films, including increased PL intensity, prolonged carrier lifetimes, and extended hole diffusion lengths.

**Figure 4:**
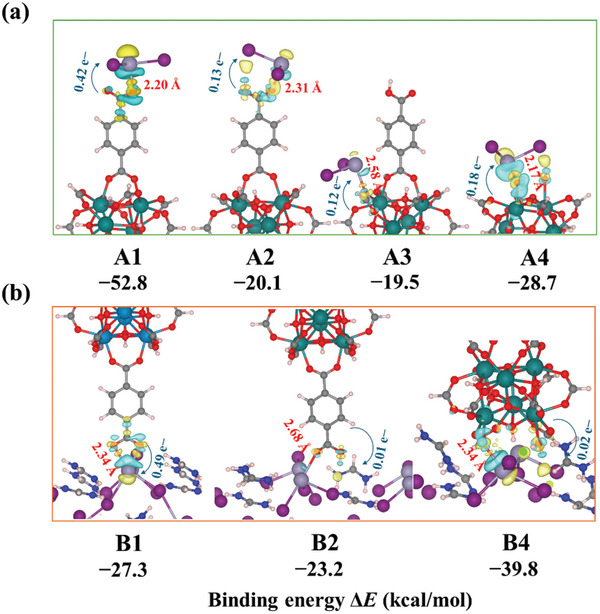
Binding interactions of SnI₂ and FASnI₃ with UiO‐66. a) Cluster model showing binding of SnI_2_ and UiO‐66 via carboxylate (A1), carboxylic (A2), bridging oxygen (A3), and hydroxyl (A4) sites with their calculated binding energies (Δ*E*), respectively. b) Periodic model of the FASnI₃ (100) surface interacting with UiO‐66 via carboxylate (B1), carboxylic (B2), and hydroxyl (B4) sites, highlighting preferred binding sites and charge transfer. Bond distances (Å) are shown in red, electrons transferred (e^−^) in blue, and binding energies (kcal mol^−1^) are noted. In the binding models, atom colors: Zr (teal), Sn (mauve), I (purple), O (red), N (dark blue), C (gray), and H (white).

Density functional theory (DFT) calculations were performed to investigate the interaction between Sn^2+^ ions and different functional groups within UiO‐66 (see **Figure** **4**a).^[^ ^43^, ^44^, ^45^ ^]^ Particularly, we examined four types of binding sites: carboxylate (─COO^−^) and carboxylic (─COOH) groups of terephthalate linkers (denoted A1 and A2), bridging oxygen atoms in Zr_6_O_4_(OH)_4_ nodes (A3), and hydroxyl groups (A4) at missing terephthalate linker defects, which commonly occur in UiO‐66.^[^ ^46^ ^]^ Computational details are provided in the Supporting Information (Tables S1–S4 and Figure S5, Supporting Information).

The calculations indicate that the interaction between Sn^2+^ and the carboxylate group is notably strongest, with an Sn─O bond distance of 2.20 Å and a binding energy of −53 kcal mol^−1^. In comparison, the Sn─OH interaction, despite a shorter bond distance of 2.17 Å, has a weaker binding energy of −28.7 kcal mol^−1^. Sn^2^⁺ binds less strongly to carboxylic and bridging oxygen sites, with Sn─O bond distances of 2.31 and 2.59 Å, and binding energies of ≈−20 kcal mol^−1^. All binding modes were thermodynamically favorable after Gibbs free energy corrections (Table S2, Supporting Information). These interactions involve electron transfer from UiO‐66 to Sn^2^⁺, with the carboxylate site transferring 0.42 electrons, more than the carboxylic (0.13), bridging oxygen (0.12), and hydroxyl (0.18) sites. The electron density difference analysis (isosurface value of 0.004 electrons/Bohr^3^) reveals electron accumulation (yellow) and depletion (blue) upon binding, indicating significant charge transfer between UiO‐66 and Sn^2^⁺.^[^ ^47^ ^]^

To model the interactions between FASnI_3_ and UiO‐66 more realistically, a periodic slab model was employed and shown in Figure 4b. Due to steric hindrance from terephthalate linkers, the bridging oxygen site was excluded. Consistent with the cluster model, the carboxylate group (B1) was found to be the preferred binding site, with a binding energy of −27.3 kcal mol^−1^ and a significant charge transfer of 0.49 electrons. In comparison, the carboxylic site (B2) exhibited a weaker binding energy of −22.3 kcal mol^−1^. The hydroxyl group (B4) also exhibited strong binding (−39.8 kcal mol^−1^), likely attributed to van der Waals interactions between the Zr₆O₄(OH)₄ nodes and the FASnI₃ surface.

These results demonstrate that Sn^2^⁺ in FASnI₃ interacts strongly with multiple sites in UiO‐66, with the carboxylate groups identified as the primary binding sites. Van der Waals interactions between the Zr₆O₄(OH)₄ nodes and the FASnI₃ surface further stabilize the interface, likely contributing to improved crystal quality and stability of the FASnI₃ films. This theoretical prediction is supported by fourier transform infrared spectroscopy (FTIR) spectrum analysis (Figure S3, Supporting Information), which shows a significant intensity change in the characteristic carboxylate peak at 1573 cm⁻¹, indicating interactions between carboxylate groups and Sn^2^⁺ ions. These interactions highlight the role of carboxylate groups in modulating the crystallization rate and promoting defect reduction in the perovskite layer.

**Figure 5:**
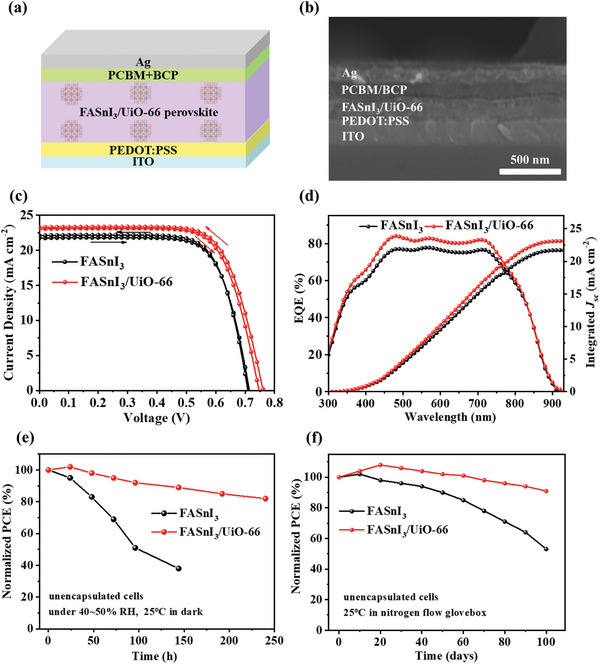
a) Schematic of TPSCs and b) cross‐section SEM micrographs with a structure of ITO/PEDOT:PSS/FAPbI_3_/UiO‐66 perovskite/PCBM/BCP/Ag. Device performance of FAPbI_3_ and FAPbI_3_/UiO‐66 based PSCs. c) current−voltage (*J−V*) curves, d) external quantum efficiency (EQE), and integrated *J*_sc_. Stability tests for unencapsulated cells in e) ambient conditions (RH of 40–50% at 25 °C, in dark) and f) a nitrogen flow glovebox (at 25 °C).

To verify the photovoltaic performance of FASnI_3_/UiO‐66 perovskite, we fabricated the p‐i‐n inverted TPSCs with a layered structure comprising ITO/PEDOT:PSS/FASnI_3_/UiO‐66 perovskite/[6,6]‐phenyl‐C_61_‐butyric acid methyl ester (PCBM)/bathocuproine (BCP)/Ag, as illustrated in **Figure** **5**a. Cross‐sectional SEM images (Figure 5b) revealed that the FASnI_3_/UiO‐66 layer has a thickness of ≈240 nm, and the small grain size of UiO‐66 (30−50 nm) ensures its thorough integration within the perovskite matrix. Additionally, a comparative SEM analysis of FASnI_3_ without UiO‐66 (Figure S4, Supporting Information) displayed a rougher surface and smaller grain size, indicating inferior crystal quality. Photovoltaic performance measured from *J–V* curves (Figure 5c and **Table** **1**) shows that the control FASnI_3_ device achieved PCE of 11.26% and 11.43%, with short‐circuit current densities (*J*_sc_) of 21.72 and 22.08 mA cm^−2^, *V*_oc_ of 0.71 and 0.70 V, and FF of 73.0% and 73.9% under forward and reverse scans, respectively. In contrast, the MOF‐integrated TPSCs demonstrated superior performance, with *J*_sc_ values of 23.04 and 23.29 mA cm^−2^, *V*_oc_ of 0.74 and 0.77 V, and slightly lower FF of 72.2% and 70.4%, culminating in higher PCEs of 12.32% and 12.64% under forward and reverse scans, respectively. The improved *J*_sc_ can be attributed to the higher absorbance, as evidenced in Figure 3a. The FASnI_3_/UiO‐66 perovskite film shows a higher external quantum efficiency (EQE) across the 350−750 nm wavelength range, as shown in Figure 5d, especially around ≈500 nm, achieving an integrated *J*_sc_ of 23.12 mA cm^−2^ compared to 21.70 mA cm^−2^ for the control device. Moreover, the increased *V*_oc_ is likely due to reduced non‐radiative recombination in FASnI_3_/UiO‐66, as indicated by the higher PL intensity and extended PL lifetime observed in Figure 3c,d. However, the lower FF is mainly due to increased series resistance (*R*_s_), which rose from 3.93 to 6.36 Ω cm^2^, attributed to the insulating properties of UiO‐66. This finding indicates that future research should focus on enhancing the charge transfer capabilities of MOF‐based perovskites to maintain lower *R_s_* and optimize overall device performance.^[^ ^35^, ^39^ ^]^

**Table 1:** Device performance parameters of FASnI_3_ and FASnI_3_/UiO‐66 based devices.

Devices	Scan direction	*J*_sc_ [mA cm^−2^]	*V*_oc_ [V]	FF [%]	PCE [%]
FASnI_3_	forward	21.72	0.71	73.0	11.26
	reverse	22.08	0.70	73.9	11.43
FASnI_3_/UiO‐66	forward	23.04	0.74	72.2	12.32
	reverse	23.29	0.77	70.4	12.64

Finally, we examined the stability of unencapsulated FASnI_3_ devices both with and without UiO‐66 under ambient conditions (humidity (RH) 40–50% at 25 °C in the dark). The PCE of the control FASnI_3_ device decreased rapidly, retaining only 38% of its initial value after 144 h, as depicted in Figure 5e. In contrast, the FASnI_3_/UiO‐66‐based device maintained over 80% of its initial PCE for 240 h. More importantly, stability tests conducted in a nitrogen flow glovebox revealed that the unencapsulated UiO‐66‐FASnI_3_ device preserved over 90% of its initial PCE for 100 days, significantly outperforming the control device, which maintained only 53% of its PCE. These findings are detailed in Figure 5f. We attribute the stability improvements primarily to the superior crystal quality of FASnI_3_/UiO‐66‐based perovskite. Additionally, we speculate that the porous structure of UiO‐66 may extend the pathway for moisture and oxygen penetration and act as a physical barrier by adsorbing these elements, potentially slowing the degradation of the perovskite.

## 3 Conclusion

This study demonstrates that UiO‐66, a zirconium‐based MOF, significantly enhances the stability and performance of TPSCs by improving the crystallinity and film quality of FASnI₃. Theoretical investigations reveal that carboxylate groups in UiO‐66 provide strong binding sites for Sn^2^⁺ ions, while van der Waals interactions further stabilize the interface. These interactions reduce defect density, enhance PL intensity, and prolong TRPL lifetimes. The resulting improvement in optical and charge transport properties leads to an increased PCE from 11.43% to 12.64%. Furthermore, UiO‐66‐integrated TPSCs exhibit remarkable stability, maintaining over 90% of their initial PCE after 100 days in a nitrogen glovebox. These findings underscore the potential of UiO‐66 to address efficiency and stability challenges and pave the way for advancements in lead‐free perovskite solar cells.

## 4 Experimental Section

### Materials

Formamidinium iodide (FAI), tin(II) iodide (SnI_2_), tin(II) fluoride (SnF_2_), BCP, zirconium tetrachloride (ZrCl_4_), terephthalic acid (H_2_BDC), and acetic acid were purchased from Aladdin. Dimethylformamide (DMF), dimethyl sulfoxide (DMSO), chlorobenzene, acetone, and methanol isopropanol (IPA) were purchased from Sigma–Aldrich. ITO glass substrates and PCBM were purchased from Xi'an Polymer Light Technology Corp. PEDOT:PSS is Clevious P VP Al 4083. All chemicals were used as received without further treatment.

### MOF Fabrication

The UiO‐66 material was synthesized with slight modifications based on established procedures in the literature.^[^ ^48^ ^]^ To prepare UiO‐66, 46.6 mg of zirconium tetrachloride was dissolved in 10 mL of DMF. Separately, 60 mg of terephthalic acid was dissolved in 20 mL of DMF with 2.5 mL of acetic acid. The zirconium tetrachloride solution was then combined with the terephthalic acid solution in a PTFE container. This container was sealed and heated in a reactor at 120 °C for 12 h. After the reaction, the UiO‐66 samples were collected by centrifugation and washed three times with DMF and methanol. The collected samples were then dried at 80 °C for 12 h and subsequently ground to obtain the final UiO‐66 product. To modulate the size of the UiO‐66 crystals, acetic acid was used in the synthesis process. By adding acetic acid, it aimed to adjust the nucleation period of crystallization, thereby producing crystals with a narrow size distribution.

### Device Fabrication

The patterned ITO glass substrates were cleaned via sequential ultra‐sonication in detergent, deionized water, acetone, and isopropanol, and finally dried in the air. Then the ITO substrates were treated with ultraviolet‐ozone for 30 min before deposition. The PEDOT:PSS solution was spin‐coated onto the ITO substrate at 4000 rpm for 30 s and then annealed at 150 °C for 10 min. After that, the substrates were immediately transferred into the nitrogen‐filled glovebox. For the deposition of tin halide perovskite film, the perovskite precursor solution composed of 0.9 m SnI_2_, 0.9 m FAI, and 0.09 m SnF_2_ in solvent DMSO was stirred for 2 h at room temperature. For FASnI_3_/UiO‐66, UiO‐66 is first dispersed in DMSO and then 0.1 mg in 1 mL of the above (0.9 m) solution. The dissolved solution was filtered with the PTFE filter (0.45 µm). Then, the mixture solution was spin‐coated onto the PEDOT:PSS layer at 1000 rpm for 10 s and at 5000 rpm for 50 s, 150 µL chlorobenzene was dripped onto the perovskite film after 30 s of the second process. Then the FASnI_3_/UiO‐66 perovskite film was annealed at 100 °C hot plate for 10 min. Afterward, 20 mg mL^−1^ PCBM/chlorobenzene solution was spin‐coated on FASnI_3_/UiO‐66 perovskite film at 1000 rpm for 60 s, then the 0.5 mg mL^−1^ BCP/IPA solution was spin‐coated on PCBM at 3000 rpm for 60 s. Finally, a 100 nm Ag electrode was evaporated on the perovskite layer under a vacuum level of 10^−4^ Pa. The active area of the device was defined as 0.09 cm^2^ with a black metal mask.

### Characterization

The morphology of the films and devices was observed by the SEM images, measured on the Hitachi SU70 field‐emission SEM. The XRD was measured by Bruker X‐ray diffractometer using Cu K_*a*_ radiation (*λ* = 1.54050 Å). The UV−vis spectra were measured by a Shimadzu UV−vis 3600 spectrophotometer. The steady‐state PL and TRPL were measured by a Hamamatsu fluorescence lifetime spectrometer. FTIR was measured by Shimadzu Irtracer‐100. The *J–V* curves were measured by a solar simulator with standard air mass AM 1.5 sunlight (100 mW cm^−2^) under reverse scan (0.8 to −0.1 V) by a fixed step voltage of 10 mV and delay time of 50 ms. The light intensity of the solar simulator was calibrated by a standard silicon solar cell. The external quantum efficiency (EQE) spectra were measured with monochromatic incident light (1 × 10^16^ photons cm^−2^) in director current mode.

### DFT Calculations

Density functional theory (DFT) calculations were conducted to investigate the interactions between UiO‐66 and FASnI₃. The Vienna Ab initio Simulation Package (VASP) was employed for geometry optimization using the PBE‐D3(BJ) functional and the projector‐augmented wave (PAW) method with a kinetic energy cutoff of 450 eV. The optimized lattice parameters for UiO‐66 and FASnI₃ showed good agreement with experimental values (Table S1, Supporting Information). Both cluster and periodic models were used to evaluate binding energies and charge transfer. The cluster model represented the UiO‐66 node as a Zr_6_O_4_(OH)_4_ unit with varying linkers, while the periodic model used a slab of the FASnI_3_ (100) surface. Binding energies (Δ*E*) and charge transfer were calculated to quantify the interactions between Sn^2^⁺ and UiO‐66. Detailed calculation methods, parameters, and results are provided in the Supporting Information (Tables S1–S4 and Figure S3, Supporting Information).

## Conflict of Interest

The authors declare no conflict of interest.

## Supporting information



Supporting Information

## Data Availability

The data that support the findings of this study are available from the corresponding author upon reasonable request.
