# Supplementary material for: Enhanced Efficiency and Stability of Tin Halide Perovskite Solar Cells Through MOF Integration
Source: Small. 2025 Jan 26;21(10):2411346. doi: 10.1002/smll.202411346 (PMC11899508; doi:10.1002/smll.202411346)
Supplement: Supplementary file 1 — Supporting Information [file SMLL-21-2411346-s001.docx]

Supporting Information

**Enhanced Efficiency and Stability of Tin Halide Perovskite Solar Cells through MOF Integration**

*Yongqi Yin*^+,^*, *Xisheng Zhang*^+^, *Ho Ngoc Nam**, *Quan Manh Phung*, *Kuina Yuan*, *Boyuan Li*, *Fanyue Kong*, *Azhar Alowasheeira*, *Baoning Wang*, *Lin Li**, *and Yusuke Yamauchi**

Prof. Y. Yin, Mr. X. Zhang, Ms. K. Yuan, Mr. B. Li, Mr. F. Kong, Dr. B. Wang, Prof. L. Li

Key Laboratory for Photonic and Electronic Bandgap Materials, Ministry of Education, School of Physics and Electronic Engineering, Harbin Normal University, Harbin 150025, China

Email: [yyq@hrbnu.edu.cn](mailto:yyq@hrbnu.edu.cn), [lil@hrbnu.edu.cn](mailto:lil@hrbnu.edu.cn)

Prof. Y. Yin, Prof. H. N. Nam, Prof. A. Alowasheeira, Prof. Y. Yamauchi,

Department of Materials Process Engineering, Graduate School of Engineering, Nagoya University, Furo-cho, Chikusa-ku, Nagoya 464–8603, Japan

Email: [honam@nagoya-u.jp](mailto:honam@nagoya-u.jp), [y.yamauchi@uq.edu.au](mailto:y.yamauchi@uq.edu.au)

Prof. Q. M. Phung

Department of Chemistry, Graduate School of Science, Nagoya University, Furo-cho, Chikusa-ku, Nagoya 464-8602, Japan.

Institute of Transformative Bio-Molecules (WPI-ITbM), Nagoya University, Furo-cho, Chikusa-ku, Nagoya 464-8601, Japan.

Prof. Y. Yamauchi

Australian Institute for Bioengineering and Nanotechnology (AIBN), The University of Queensland, Brisbane, Queensland 4072, Australia

Prof. Y. Yamauchi

Department of Plant & Environmental New Resources and Graduate School of Green-Bio Science, Kyung Hee University, 1732 Deogyeong-daero, Giheung-gu, Yongin-si, Gyeonggi-do 17104, South Korea

**
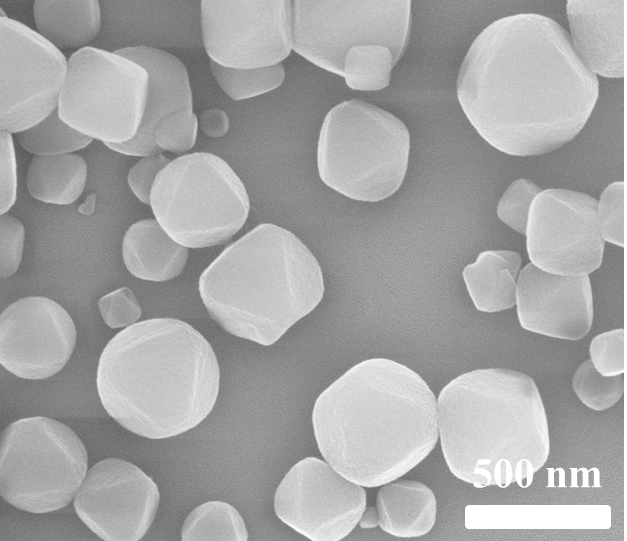
**

Figure S1. SEM images UiO-66 with 300-500 nm grain size


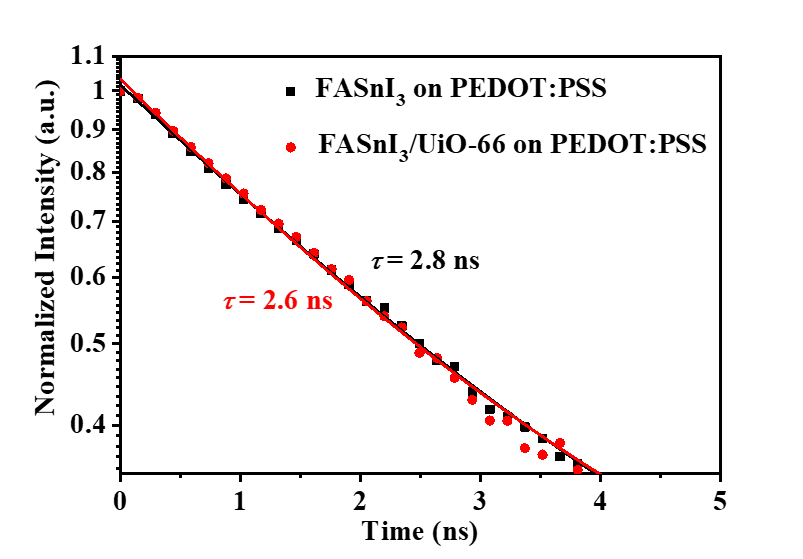


**Figure S2.** The TRPL lifetime of FASnI_3_ and FASnI_3_/UiO-66 perovskite films on PEDOT:PSS


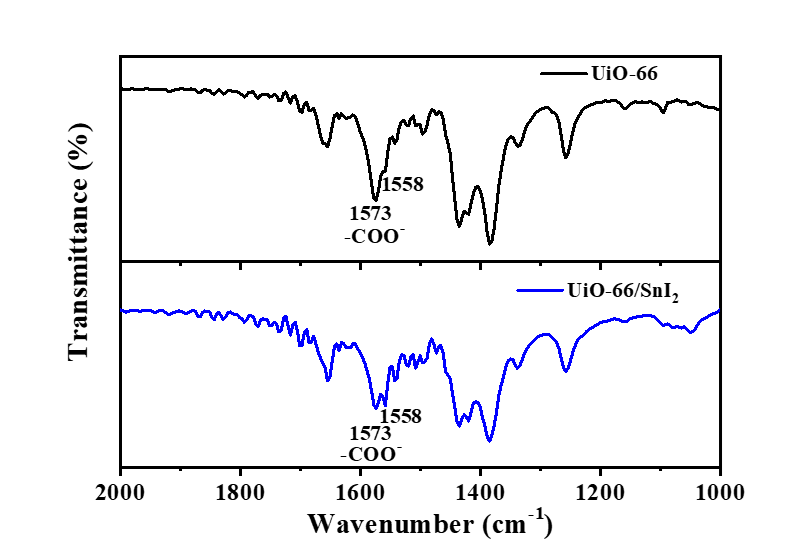


Figure S3. Fourier transform infrared spectroscopy of UiO-66 and UiO-66/SnI_2_


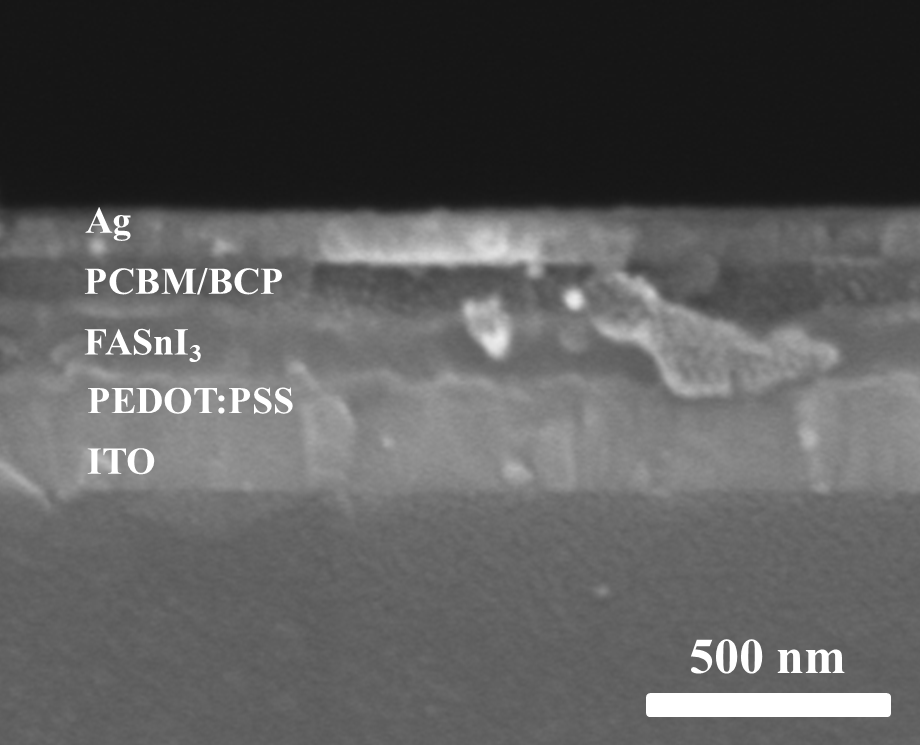


Figure S4. Cross-sectional SEM image of a perovskite solar cell without UiO-66

**DFT calculations**

The crystal structures of UiO-66 and FASnI_3_ were optimized using the Vienna Ab initio Simulation Package (VASP)^[1,2]^ with the PBE-D3(BJ)^[3]^ functional. We used the projector-augmented wave (PAW) method^[4,5]^ with a kinetic energy cutoff of 450 eV. The optimized lattice parameters are shown in **Table S1.**

**Table S1.** Calculated lattice parameters (Å) of UiO-66 and FASnI_3_.

|  | UiO-66 | | FASnI_3_ | |
| --- | --- | --- | --- | --- |
|  | This work | Exp.^[6]^ | This work | DFT^[7]^ |
| *a* (Å) | 20.823 | 20.7465 | 8.793 | 8.99 |
| *b* (Å) | 20.823 | 20.7465 | 8.793 | 9.19 |
| *c* (Å) | 20.823 | 20.7465 | 12.815 | 12.63 |

To study the binding interactions between Sn²⁺ ions and UiO-66, both cluster and periodic calculations were performed.

1. **Cluster calculations:**

The UiO-66 node was modeled as a Zr₆O₄(OH)₄ unit with one complete terephthalate linker and 11 formate anions replacing the remaining linkers (**MOF1** and **MOF2**, Figure S5). Additionally, a defect model (**MOF3**) was considered, where one linker was replaced by an OH/H₂O pair. All geometries were obtained at the PBE-D3(BJ) level, with def2-TZVP basis set for I, Sn, and Zr atoms; and def2-SVP basis set for all other atoms (denoted as BS1).^[8]^ We confirmed the nature of the optimized structures through frequency analysis and obtained quasi-harmonic free energy corrections using a cutoff of 100 cm^−1^.^[9,10]^ Single-point calculations were then carried out on the optimized structures using the PBE-D3(BJ)/def2-TZVP and B3LYP-D3(BJ)/def2-TZVP levels.^[11–14]^ The binding energy of SnI_2_ to MOF was calculated as:

Δ*E* = *E*(SnI_2_-MOF) – *E*(SnI_2_) – *E*(MOF)

The binding free energy was also calculated and reported in **Table S2**. All cluster calculations were performed using Gaussian 16.^[15]^ All raw DFT data are provided in **Tables S3** and **S4**. For electron density analysis, the isosurface value was set at 0.004 electrons/Bohr³ to visualize electron density changes. Yellow regions indicate electron accumulation, while blue regions indicate electron depletion upon binding, revealing significant charge transfer between UiO-66 and Sn²⁺ ions.

**Electron Density Analysis**:
The isosurface value was set at 0.004 electrons/Bohr³ to visualize electron density changes. Yellow regions indicate electron accumulation, while blue regions indicate electron depletion upon binding, revealing significant charge transfer between UiO-66 and Sn²⁺ ions.

1. **Periodic Calculations:**

For the periodic calculations, a slab of FASnI_3_(100) surface (**Figure S5**) was used along with the same Zr_6_O_4_(OH)_4_ nodes. These calculations employed the PBE-D3(BJ)^[3]^ functional with a 450 eV kinetic cutoff and Gamma-point sampling. During geometry optimization, the bottom atoms of the FASnI_3_(100) surface were kept fixed. The binding energy between the MOF cluster and FASnI_3_(100) was calculated as:

Δ*E* = *E*(FASnI_3_-MOF) – *E*(FASnI_3_) – *E*(MOF)

Charge transfer between Sn^2+^ and MOF was analyzed using the chargemol program.^[16]^


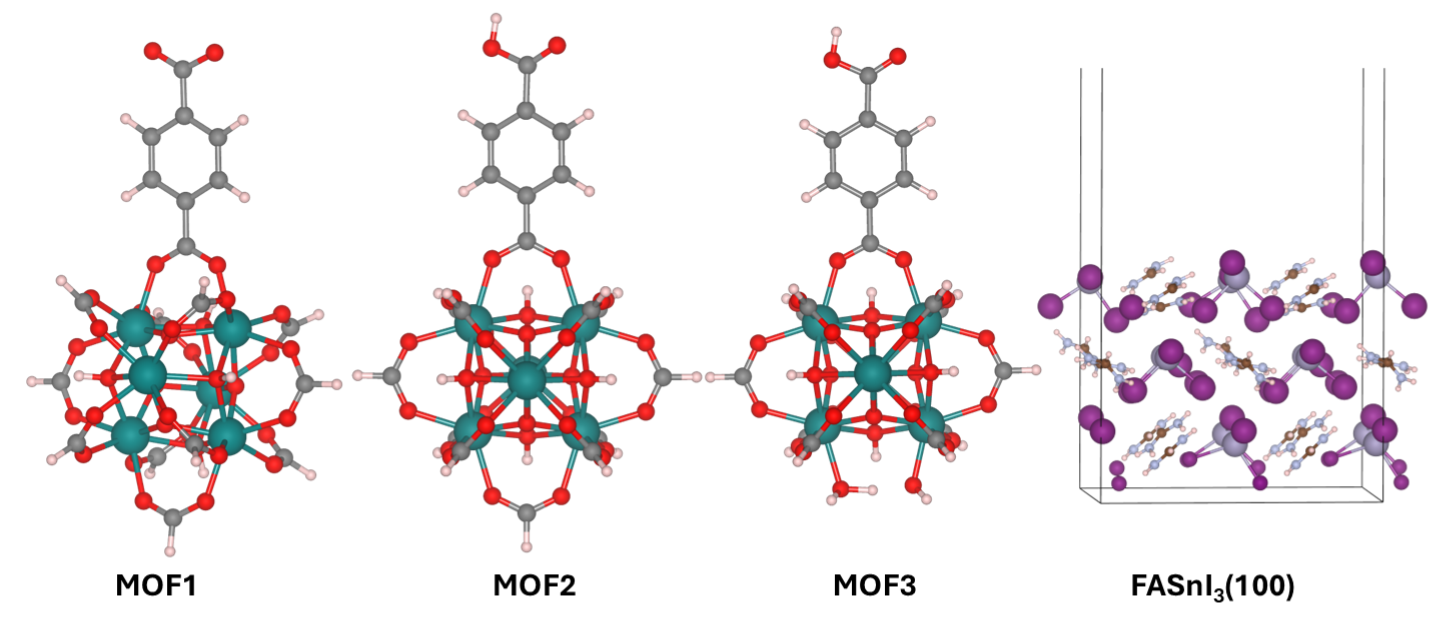


**Figure S5**. Models used in this work. **MOF1** and **MOF2**: Zr_6_O_4_(OH)_4_ node with one terephthalate linker and 11 formate caps. **MOF3**: Zr_6_O_4_(OH)_4_ node with one missing linker, substituted by an OH/H_2_O pair. **FASnI_3_(100)** surface: 8 bottom iodine atoms (shown smaller in size) are fixed during optimization.

**Table S2.** Binding energies and binding free energies (in kcal/mol), calculated for the cluster models with PBE-D3(BJ)/def2-TZVP and B3LYP-D3(BJ)/def2-TZVP levels.

|  | Δ*E* (PBE) | Δ*E* (B3LYP) | Δ*G* (PBE) | Δ*G* (B3LYP)*^a^* |
| --- | --- | --- | --- | --- |
| **A1** | -49.4 | -52.8 | -32.9 | -36.3 |
| **A2** | -19.6 | -20.1 | -4.7 | -5.2 |
| **A3** | -16.0 | -19.5 | -0.3 | -3.8 |
| **A4** | -23.6 | -28.7 | -8.3 | -13.4 |
| *^a^*Gibbs free energy corrections taken from calculations with PBE. | | | | |

**Table S3.** Raw energies (in *E*_h_) of species in the cluster calculations, calculated with PBE-D3(BJ)/BS1, PBE-D3(BJ)/def2-TZVP, and B3LYP-D3(BJ)/def2-TZVP.

|  | *E* (PBE/BS1) | *G* (PBE/BS1)*^a^* | *E* (PBE/def2-TZVP) | *E* (B3LYP/def2-TZVP) |
| --- | --- | --- | --- | --- |
| SnI_2_ | -809.820364 | -809.853072 | -809.820364 | -810.112909 |
| **MOF1** | -3572.165482 | -3571.806758 | -3575.816477 | -3579.415926 |
| **MOF2** | -3572.710572 | -3572.33672 | -3576.352153 | -3579.958698 |
| **MOF3** | -3535.866204 | -3535.479832 | -3539.473028 | -3543.043146 |
| **A1** | -4382.078972 | -4381.726726 | -4385.715514 | -4389.612947 |
| **A2** | -4382.569484 | -4382.204632 | -4386.203718 | -4390.103650 |
| **A3** | -4382.559658 | -4382.193437 | -4386.197993 | -4390.102752 |
| **A4** | -4345.728160 | -4345.350005 | -4349.331056 | -4353.201866 |
| *^a^*Gibbs free energy corrections calculated with quasi-harmonic approximation proposed by Grimme.^[9]^ | | | | |

**Table S4.** Raw energies (in eV) of species in the slab model, calculated with PBE-D3(BJ).

|  | *E* (PBE) |
| --- | --- |
| FASnI_3_(100) | -676.370278 |
| **MOF1** | -572.054996 |
| **MOF2** | -574.156901 |
| **MOF3** | -572.910378 |
| **B1** | -1249.608172 |
| **B2** | -1251.533753 |
| **B4** | -1251.007546 |

**References**

[1] G. Kresse, J. Furthmüller, *Comput Mater Sci* **1996**, *6*, 15–50.

[2] G. Kresse, J. Hafner, *Phys Rev B* **1993**, *47*, 558–561.

[3] J. P. Perdew, K. Burke, M. Ernzerhof, *Phys Rev Lett* **1996**, *77*, 3865–3868.

[4] P. E. Blöchl, *Phys Rev B* **1994**, *50*, 17953–17979.

[5] G. Kresse, D. Joubert, *Phys Rev B* **1999**, *59*, 1758–1775.

[6] S. Øien, D. Wragg, H. Reinsch, S. Svelle, S. Bordiga, C. Lamberti, K. P. Lillerud, *Cryst Growth Des* **2014**, *14*, DOI 10.1021/cg501386j.

[7] L. Peng, W. Xie, *RSC Adv* **2020**, *10*, DOI 10.1039/d0ra02584d.

[8] F. Weigend, R. Ahlrichs, *Physical Chemistry Chemical Physics* **2005**, *7*, 3297–3305.

[9] S. Grimme, *Chemistry - A European Journal* **2012**, *18*, 9955–9964.

[10] G. Luchini, J. V. Alegre-Requena, I. Funes-Ardoiz, R. S. Paton, *F1000Res* **2020**, *9*, 291.

[11] A. D. Becke, *J Chem Phys* **1993**, *98*, 5648–5652.

[12] C. Lee, W. Yang, R. G. Parr, *Phys Rev B* **1988**, *37*, 785–789.

[13] S. H. Vosko, L. Wilk, M. Nusair, *Can J Phys* **1980**, *58*, 1200–1211.

[14] P. J. Stephens, F. J. Devlin, C. F. Chabalowski, M. J. Frisch, *J Phys Chem* **1994**, *98*, 11623–11627.

[15] M. J. Frisch, G. W. Trucks, H. B. Schlegel, G. E. Scuseria, M. A. Robb, J. R. Cheeseman, G. Scalmani, V. Barone, G. A. Petersson, H. Nakatsuji, *Gaussian Inc: Wallingford, CT, USA* **2016**.

[16] T. A. Manz, N. G. Limas, *RSC Adv* **2016**, *6*, 47771–47801.

**Cartesian coordinates**

**SnI_2_**

Sn 0.000000 1.190976 0.000000

I 2.126208 -0.561782 0.000000

I -2.126208 -0.561780 0.000000

**MOF1**

O 4.722667 -1.086278 0.476940

O 2.019787 -0.845196 3.818807

O 3.254345 2.756323 1.782812

O 2.021208 1.827493 3.456873

O 3.252262 -2.185737 2.451349

O 4.723835 1.171144 0.171438

C 5.271418 0.054851 0.418054

C 2.925271 -1.697125 3.574971

C 2.927076 2.583751 2.995773

O 2.222826 0.152874 1.135060

H 0.677587 3.023822 1.576956

O 0.721681 2.161463 1.129117

H 3.728481 -0.239060 -1.758791

O 2.896425 -0.171391 -1.259823

O -0.326923 3.874690 -0.655889

O -0.272348 0.931961 -3.830641

O 3.431200 2.315327 -2.124795

O 2.412607 0.958482 -3.643602

O -1.504892 2.277962 -2.470894

O 1.946489 3.890913 -0.514634

C 0.811638 4.424349 -0.714379

C -1.107501 1.859748 -3.597393

C 3.186988 1.899568 -3.297921

O 0.884198 1.320150 -1.227378

Zr 2.570362 1.772545 -0.120858

H 0.675636 -2.496008 2.323612

O 0.720199 -1.783714 1.662695

H -7.830094 -2.132487 0.232194

C -7.238611 -1.210359 0.122517

O -10.050938 -1.064168 -0.325576

H -5.280073 2.131043 -0.426328

C -5.846805 1.200961 -0.268227

O -3.035053 1.095749 -0.345981

O -0.848423 1.799430 3.276391

O -1.875851 -2.230808 2.134479

O -0.849539 -0.863847 3.636304

O -1.873621 2.718744 1.465068

H -7.828286 2.129116 -0.267480

C -7.238972 1.201523 -0.200389

O -10.015003 1.080423 0.441181

H -5.277135 -2.157528 0.211142

C -5.845206 -1.222553 0.091448

O -3.035470 -1.146147 -0.044092

C -3.666322 -0.021218 -0.166781

C -9.495574 0.005700 0.039380

C -1.710603 2.539666 2.707404

C -1.712142 -1.728162 3.284527

C -7.957654 -0.001163 -0.013807

C -5.127096 -0.014210 -0.114665

O -0.711707 0.127532 0.938355

Zr 0.641102 0.336024 2.484682

O 1.942740 -3.888714 0.537638

O 2.411541 -1.892947 -3.257865

O -1.505602 -2.850743 -1.777252

O -0.273351 -1.914511 -3.445986

O 3.428746 -2.798821 -1.433026

O -0.330701 -3.909259 0.397163

C 0.807535 -4.455359 0.487056

C 3.184913 -2.709267 -2.674508

C -1.108612 -2.746913 -2.974495

O 0.883118 -1.598868 -0.832698

Zr 2.568977 -1.742555 0.354621

H -1.854563 -0.282946 -2.097902

O -1.063425 -0.206715 -1.535429

Zr -0.985746 1.738638 -0.351924

Zr 0.978875 -0.335449 -2.480300

Zr -0.986954 -1.768265 0.122166

H -2.397018 3.107537 3.385750

H 0.820919 5.517492 -0.962176

H -2.398494 -2.095117 4.089609

H 6.378357 0.078280 0.595381

H 3.500920 -2.072231 4.460833

H 3.502872 3.180302 3.750362

H -1.549904 2.360789 -4.496415

H 3.730712 2.421565 -4.127743

H 0.816116 -5.575033 0.539090

H 3.728034 -3.433455 -3.335781

H -1.551379 -3.468621 -3.707997

**MOF2**

O -4.730113 1.152310 0.376306

O -1.976289 1.361096 3.677738

O -3.249527 -2.469342 2.175440

O -1.985517 -1.330936 3.685343

O -3.232473 2.499490 2.161275

O -4.737921 -1.122203 0.382747

C -5.286031 0.017217 0.479561

C -2.889062 2.173057 3.337554

C -2.903776 -2.138674 3.349834

O -2.216678 0.008645 1.157090

H -0.683970 -2.780731 1.973895

O -0.726251 -1.987494 1.411825

H -3.767139 0.005630 -1.739011

O -2.929530 0.004225 -1.243647

O 0.286609 -3.923564 -0.142924

O 0.219084 -1.451613 -3.666371

O -3.472591 -2.570071 -1.754528

O -2.464017 -1.441500 -3.452446

O 1.480131 -2.587441 -2.151823

O -1.980881 -3.915793 0.037989

C -0.853358 -4.477956 -0.106016

C 1.058680 -2.337103 -3.321493

C -3.240506 -2.324040 -2.976753

O -0.920553 -1.474255 -1.032431

Zr -2.607606 -1.769207 0.148198

H -0.665085 2.792089 1.958081

O -0.712558 1.996002 1.400493

H 7.813434 2.140520 -0.026151

C 7.252085 1.196554 -0.051131

O 10.020873 1.139067 0.082024

H 5.275726 -2.182128 -0.134946

C 5.848306 -1.243975 -0.111751

O 3.058766 -1.145714 -0.241784

O 0.881285 -1.341083 3.456497

O 1.902193 2.480709 1.753698

O 0.890327 1.350689 3.449010

O 1.884912 -2.487727 1.767597

H 7.817161 -2.183971 -0.014724

C 7.243542 -1.246255 -0.045158

O 10.093045 -1.121778 0.091088

H 5.287664 2.139862 -0.145757

C 5.855333 1.198833 -0.117854

O 3.066371 1.119341 -0.248376

C 3.651494 -0.015172 -0.215142

C 9.443732 -0.090443 0.057318

C 1.731888 -2.152673 2.981203

C 1.746515 2.153527 2.969146

C 7.952136 -0.027551 -0.014581

C 5.147599 -0.020207 -0.149117

O 0.717415 -0.002000 0.924849

Zr -0.632560 0.006988 2.506556

O -1.953889 3.925079 0.015685

O -2.454103 1.434226 -3.460587

O 1.497882 2.560775 -2.166624

O 0.228966 1.424801 -3.674579

O -3.454933 2.579308 -1.769126

O 0.313684 3.916427 -0.165095

C -0.822585 4.478633 -0.131471

C -3.224556 2.324688 -2.989925

C 1.074521 2.306470 -3.334782

O -0.910471 1.470233 -1.040878

Zr -2.595436 1.783152 0.138104

H 1.795670 -0.014678 -2.171724

O 1.037245 -0.010179 -1.561948

Zr 0.932675 -1.779372 -0.134777

Zr -1.032325 -0.005971 -2.503245

Zr 0.944655 1.767403 -0.144854

H 2.420560 -2.636654 3.719419

H -0.864952 -5.593398 -0.201537

H 2.438403 2.636924 3.704809

H -6.389660 0.021549 0.667853

H -3.449592 2.666025 4.171973

H -3.467461 -2.623176 4.187068

H 1.484167 -2.963778 -4.145939

H -3.792427 -2.951628 -3.721712

H -0.826633 5.593593 -0.233432

H -3.772251 2.951749 -3.738454

H 1.504137 2.925499 -4.162906

H 10.986408 0.969600 0.129010

**MOF3**

O 4.785247 -1.221138 0.347576

O 2.003088 -1.412108 3.679355

O 3.310043 2.436919 2.255180

O 2.001139 1.286851 3.719915

O 3.326225 -2.481866 2.167034

O 4.724868 1.267879 0.312707

H 5.164560 1.712621 1.061289

C 2.940452 -2.190302 3.344979

C 2.928993 2.095038 3.415773

O 2.251126 -0.024546 1.167339

H 0.707507 2.757934 2.006228

O 0.768335 1.971525 1.436598

H 3.861708 0.023502 -1.690138

O 3.009502 0.002101 -1.216540

O -0.234345 3.921906 -0.115752

O -0.185866 1.470907 -3.659318

O 3.394041 2.657787 -1.779524

O 2.491836 1.428579 -3.463666

O -1.437499 2.601600 -2.132805

O 2.026583 3.929185 0.134677

C 0.898973 4.484543 -0.033351

C -1.021608 2.355674 -3.306030

C 3.204942 2.371581 -2.993917

O 0.961234 1.463841 -1.024276

Zr 2.662363 1.783150 0.170484

H 0.688599 -2.814515 1.945490

O 0.751170 -2.015804 1.393419

H -7.778540 -2.128966 -0.045345

C -7.214739 -1.186287 -0.062534

O -9.983334 -1.123018 0.072020

H -5.229328 2.187559 -0.119560

C -5.804666 1.250920 -0.103583

O -3.015557 1.146291 -0.237041

O -0.857532 1.307095 3.460491

O -1.867019 -2.501303 1.729930

O -0.859025 -1.385159 3.436860

O -1.843731 2.476187 1.776846

H -7.770974 2.195282 0.002269

C -7.199872 1.256325 -0.036191

O -10.050023 1.137621 0.100766

H -5.252644 -2.133715 -0.166128

C -5.818023 -1.191589 -0.129926

O -3.029455 -1.119091 -0.259426

C -3.610732 0.017995 -0.218136

C -9.402899 0.105180 0.057804

C -1.702553 2.126025 2.987225

C -1.714983 -2.182680 2.948331

C -7.911678 0.039274 -0.015306

C -5.107094 0.025774 -0.151319

O -0.683441 -0.011702 0.922900

Zr 0.663163 -0.029557 2.500224

O 1.987868 -3.938589 0.032351

O 2.490618 -1.416388 -3.464530

O -1.458781 -2.548621 -2.186838

O -0.189763 -1.400745 -3.686605

O 3.447234 -2.607719 -1.782346

O -0.275379 -3.921432 -0.195136

C 0.856754 -4.488183 -0.137776

C 3.241033 -2.326580 -3.000505

C -1.034351 -2.284418 -3.353498

O 0.946860 -1.465995 -1.052746

Zr 2.613132 -1.790372 0.124281

H -1.758479 0.026229 -2.174304

O -1.000074 0.015832 -1.564743

Zr -0.882012 1.776346 -0.129365

Zr 1.079740 0.023252 -2.505163

Zr -0.910981 -1.768636 -0.162939

H -2.396964 2.602162 3.725413

H 0.903436 5.601852 -0.106063

H -2.410395 -2.670505 3.677892

H 3.496887 -2.687288 4.180880

H 3.471969 2.566554 4.275003

H -1.450278 2.987717 -4.124831

H 3.728845 3.011646 -3.748764

H 0.859151 -5.603364 -0.237828

H 3.790127 -2.947703 -3.752990

H -1.464257 -2.898119 -4.185581

H 5.097765 -1.554304 1.210735

H 4.856139 -0.135487 0.375169

H -10.948247 -0.951151 0.120629

**A1**

O 7.206521 -0.924369 0.126071

O 4.248852 -3.390145 2.052164

O 5.140500 0.526110 3.621119

O 3.917839 -1.383270 3.819296

O 5.753414 -3.181011 0.357216

O 6.926205 0.771081 1.619257

C 7.601260 -0.122209 1.024921

C 5.278271 -3.730948 1.396525

C 4.747391 -0.516349 4.226939

O 4.516043 -0.696404 1.098226

H 2.574012 0.781514 3.302871

O 2.766915 0.564441 2.374252

H 6.275991 1.295548 -0.833630

O 5.408144 0.933056 -0.585166

O 1.641665 3.000547 2.340938

O 2.312762 3.501243 -1.892507

O 5.664940 3.218822 0.796126

O 4.949093 3.461758 -1.350705

O 0.807402 3.294626 -0.199580

O 3.872135 2.971179 2.792659

C 2.695418 3.436670 2.891562

C 1.342799 3.897665 -1.176170

C 5.563917 3.835391 -0.307424

O 3.217768 1.813316 0.248765

Zr 4.745924 1.318520 1.558798

H 3.260565 -3.368706 -0.350609

O 3.257401 -2.402381 -0.237605

H -5.058074 -1.968731 -2.531262

C -4.588711 -1.223411 -1.872340

O -7.405219 -1.393301 -2.049733

H -3.057993 1.461748 0.525678

C -3.504912 0.714282 -0.145741

O -0.712752 0.874030 0.054729

O 1.099793 -1.346901 3.244973

O 0.713360 -3.118813 -0.663390

O 1.430625 -3.350094 1.482165

O 0.100071 0.591344 2.601645

H -5.583661 1.239288 0.286850

C -4.891428 0.595516 -0.274653

O -7.645337 0.311093 -0.586438

H -2.522534 -1.766015 -2.310205

C -3.202636 -1.109604 -1.748153

O -0.434886 -0.809918 -1.426315

C -1.180406 -0.018930 -0.741160

C -6.941327 -0.522343 -1.292270

C 0.190333 -0.462259 3.299427

C 0.720465 -3.667282 0.478773

C -5.440673 -0.372632 -1.139714

C -2.650195 -0.137148 -0.882725

O 1.634638 -0.654752 0.509595

Zr 2.832614 -1.655192 1.871573

O 4.838057 -2.872712 -2.351803

O 5.303013 1.319601 -3.236708

O 1.441637 -0.547755 -3.582102

O 2.666519 1.359473 -3.777618

O 6.299175 -0.619960 -2.583785

O 2.607374 -2.844496 -2.804233

C 3.798595 -3.235681 -2.982984

C 6.136233 0.372328 -3.356434

C 1.914041 0.438801 -4.220843

O 3.580267 -0.380474 -1.682624

Zr 5.182812 -1.324580 -0.768135

H 0.795657 1.355341 -1.930188

O 1.515964 0.992775 -1.384761

Zr 1.261296 1.364703 0.848187

Zr 3.627325 1.696394 -1.786162

Zr 1.698012 -1.274834 -1.475088

H -0.616408 -0.627292 4.057331

H 2.574230 4.333070 3.552244

H 0.030367 -4.537749 0.616288

H 8.678482 -0.205960 1.322167

H 5.828829 -4.630869 1.773862

H 5.177401 -0.685568 5.247576

H 0.904662 4.892485 -1.444906

H 6.089637 4.822798 -0.370591

H 3.952859 -4.000681 -3.786770

H 6.817225 0.422648 -4.244782

H 1.627871 0.512530 -5.301049

Sn -9.780266 -0.108875 -0.939815

I -10.436387 2.321695 0.510030

I -9.890121 -2.031414 1.196097

**A2**

O 7.138796 0.181958 1.212128

O 4.115106 2.725643 2.920403

O 5.357565 3.300410 -1.161331

O 4.012435 3.980734 0.541638

O 5.546468 0.985195 3.227488

O 7.052276 1.242088 -0.798049

C 7.636930 0.851939 0.257539

C 5.087457 2.138991 3.485012

C 4.923451 4.148689 -0.324510

O 4.519791 1.154959 0.551914

H 2.809448 3.019413 -1.543643

O 2.933113 2.160699 -1.102785

H 6.318263 -1.244333 -0.791178

O 5.441611 -0.887327 -0.565128

O 1.983547 1.590412 -3.544043

O 2.457353 -2.651328 -2.994508

O 5.920439 -0.092482 -3.079079

O 5.110155 -2.207718 -2.874066

O 1.021864 -0.913207 -3.299050

O 4.224540 1.965756 -3.442784

C 3.092203 1.990767 -4.012692

C 1.555441 -2.018159 -3.621361

C 5.805712 -1.305295 -3.431199

O 3.362566 -0.211122 -1.785427

Zr 4.927509 1.129482 -1.488820

H 3.021587 0.421040 3.381943

O 3.084756 0.303454 2.417977

H -5.276085 -1.799146 1.871420

C -4.752565 -1.332925 1.025575

O -7.518661 -1.347128 1.057209

H -2.915529 0.346785 -1.998400

C -3.450703 -0.122525 -1.160822

O -0.661627 -0.051964 -1.143669

O 1.179005 3.504780 0.412393

O 0.469206 0.134730 2.996789

O 1.281609 2.249325 2.791471

O 0.280219 2.449363 -1.390947

H -5.449305 0.248855 -1.943840

C -4.845749 -0.176915 -1.130126

O -7.649182 -0.376245 -0.988281

H -2.749730 -1.693591 1.807376

C -3.357057 -1.276054 0.991854

O -0.574971 -1.109602 0.859465

C -1.201238 -0.604426 -0.129361

C -6.984059 -0.822835 -0.021789

C 0.340474 3.379583 -0.530172

C 0.504561 1.372079 3.275168

C -5.502186 -0.782787 -0.037350

C -2.701317 -0.672303 -0.100468

O 1.619163 0.671223 0.421626

Zr 2.825745 2.192231 1.172057

O 4.522859 -1.689212 3.486390

O 5.219644 -3.548403 -0.332630

O 1.218501 -3.311959 1.248334

O 2.567042 -3.992363 -0.452425

O 6.116059 -2.492861 1.471531

O 2.281873 -2.064118 3.384990

C 3.433041 -2.184767 3.903923

C 5.982634 -3.472666 0.677642

C 1.732713 -4.183227 0.482876

O 3.474578 -1.584257 0.818093

Zr 5.062687 -0.526826 1.651075

H 0.821253 -2.156109 -1.035680

O 1.518858 -1.544133 -0.742823

Zr 1.429857 0.542169 -1.645858

Zr 3.663429 -2.176307 -1.168611

Zr 1.565060 -1.112535 1.490825

H -0.429386 4.187470 -0.619015

H 3.066043 2.425190 -5.044105

H -0.228105 1.728344 4.042968

H 8.719295 1.121231 0.352961

H 5.591878 2.699846 4.312124

H 5.390951 5.165163 -0.361241

H 1.179366 -2.493665 -4.562554

H 6.393633 -1.617848 -4.331108

H 3.491731 -2.789964 4.843854

H 6.618528 -4.369980 0.886534

H 1.404274 -5.241046 0.645637

H -8.526254 -1.406673 0.967564

Sn -9.823300 0.364159 -1.247094

I -9.374636 2.251598 0.766114

I -10.811576 -1.769578 0.412479

**A3**

O -4.772994 -2.692985 0.648225

O -1.892043 -3.158853 -2.489783

O -4.868049 -0.069380 -2.767627

O -3.077273 -1.085681 -3.730603

O -2.642757 -3.894625 -0.474827

O -5.787149 -0.946121 -0.396967

C -5.759778 -2.110961 0.103771

C -2.367169 -4.034419 -1.705095

C -4.288527 -0.710028 -3.695695

O -2.962670 -1.065919 -0.857500

H -2.737395 1.384566 -3.041031

O -2.486225 1.012352 -2.177564

H -4.662118 -0.411553 1.872523

O -3.862620 -0.268860 1.336193

O -2.601763 3.660995 -1.820410

O -1.976013 3.356715 2.419196

O -5.541489 1.713028 0.668046

O -4.335072 2.068958 2.561811

O -1.173903 4.081823 0.418959

O -4.601669 2.584681 -1.698210

C -3.858917 3.576341 -1.968182

C -1.576791 4.247922 1.610431

C -5.364540 2.187797 1.830634

O -2.699677 1.654955 0.231617

Zr -4.209228 0.601747 -0.738946

H -0.260998 -2.907643 -0.477557

O -0.699115 -2.047006 -0.354022

H 6.515668 2.867084 1.176876

C 5.692012 3.180173 0.520657

O 8.104823 4.538528 0.411628

H 2.748153 4.339248 -1.788449

C 3.587283 4.023842 -1.151857

O 1.090232 2.776806 -0.763985

O -0.531240 0.269325 -3.858434

O 1.938323 -1.402132 -0.852070

O 0.618786 -1.834694 -2.650724

O -0.323764 2.372642 -3.018615

H 4.985590 5.546353 -1.856053

C 4.814572 4.689803 -1.187964

O 7.362432 5.973276 -1.172921

H 4.311937 1.659405 1.238832

C 4.467780 2.503070 0.548132

O 2.091160 1.029235 0.273555

C 2.106370 2.194823 -0.265055

C 7.161995 5.024635 -0.434252

C -0.177868 1.486097 -3.914055

C 1.715869 -1.844264 -2.040953

C 5.872083 4.271339 -0.353978

C 3.412634 2.919150 -0.291925

O -0.379051 0.332850 -1.006170

Zr -1.420770 -0.990164 -2.237058

O -1.105137 -3.437444 1.904803

O -3.057595 -0.146438 3.883236

O 1.063437 0.118686 2.805205

O -0.715803 1.158755 3.759854

O -3.243247 -2.248582 3.036703

O 0.885567 -2.354463 1.744576

C 0.137269 -3.308125 2.122436

C -3.291293 -1.389588 3.969286

C 0.457052 0.673559 3.769227

O -1.395596 -0.602592 1.586953

Zr -2.623753 -2.135246 0.897688

H 0.221156 2.250274 1.607942

O -0.368515 1.632042 1.141557

Zr -1.084152 2.290621 -0.915815

Zr -2.325774 1.157248 2.217943

Zr 0.475461 -0.430766 0.726110

H 0.310368 1.817354 -4.865231

H -4.369224 4.476339 -2.395236

H 2.596251 -2.291510 -2.566192

H -6.718388 -2.687769 0.070545

H -2.554937 -5.046234 -2.144365

H -4.912636 -0.964152 -4.589195

H -1.569268 5.299181 1.994320

H -6.216907 2.768999 2.264424

H 0.633765 -4.126022 2.701184

H -3.582844 -1.771651 4.979940

H 1.021935 0.753466 4.731572

H 8.896426 5.101632 0.272261

Sn 4.459784 -0.960755 -0.493013

I 5.002788 -3.616054 -1.280078

I 4.525665 -1.287401 2.275169

**A4**

O 3.116801 2.621106 0.624270

O 0.305232 2.718410 -3.078624

O 2.115967 -1.124419 -2.765624

O 0.638941 0.188938 -3.885360

O 1.475777 3.466344 -1.280193

O 3.522443 -0.777736 -0.644079

H 3.859665 -0.729006 -1.562363

C 1.149138 3.470510 -2.505888

C 1.682351 -0.523939 -3.795979

O 0.810311 0.757499 -1.042191

H -0.386520 -1.852883 -2.674046

O -0.432149 -1.264444 -1.900073

H 2.419631 0.128250 1.757369

O 1.597130 0.176156 1.231568

O -1.161413 -3.688979 -0.992567

O -1.416689 -2.355584 3.093539

O 2.318222 -2.445821 1.096771

O 1.223352 -1.904263 3.009224

O -2.510635 -3.162024 1.271230

O 1.077129 -3.329099 -1.139579

C 0.037227 -4.055101 -1.181490

C -2.127611 -3.207253 2.479665

C 2.069891 -2.564479 2.330328

O -0.282489 -1.456363 0.605475

Zr 1.418207 -1.206458 -0.591129

H -1.137821 3.413154 -1.045864

O -0.985015 2.504003 -0.733647

H -9.443608 1.078900 0.440679

C -8.757384 0.254211 0.204544

O -11.493545 -0.140006 -0.005338

H -6.334958 -2.701836 -0.646315

C -7.030796 -1.884027 -0.410706

O -4.279473 -1.449498 -0.185515

O -2.158544 -0.268327 -3.698722

O -3.646820 2.722353 -1.001130

O -2.513610 2.273345 -2.920542

O -2.956316 -1.990454 -2.447538

H -8.853299 -3.014373 -0.823130

C -8.413342 -2.056863 -0.508693

O -11.253454 -2.288679 -0.671307

H -6.940760 1.388463 0.615891

C -7.373333 0.427587 0.302629

O -4.597465 0.693896 0.471783

C -5.023575 -0.451869 0.098434

C -10.753127 -1.232622 -0.324435

C -2.878066 -1.293847 -3.504965

C -3.462744 2.785827 -2.255229

C -9.282848 -0.990143 -0.201837

C -6.504439 -0.639985 -0.003516

O -2.122770 0.283214 -0.890173

Zr -0.814006 0.926386 -2.368506

O -0.007083 4.096778 1.165654

O 0.825276 0.740563 3.831018

O -3.226205 1.710732 2.769313

O -1.815362 0.351449 3.924154

O 1.605037 2.513323 2.648690

O -2.246982 3.733153 1.292551

C -1.200980 4.431108 1.441736

C 1.423734 1.849015 3.713186

C -2.770963 1.177786 3.827602

O -0.692906 1.320698 1.461067

Zr 0.893576 2.187102 0.472270

H -3.169684 -0.772746 2.027360

O -2.426426 -0.489345 1.466670

Zr -2.089564 -1.741007 -0.400328

Zr -0.369650 -0.494868 2.431145

Zr -2.593228 1.616950 0.630546

H -3.507761 -1.628791 -4.367460

H 0.197231 -5.136022 -1.421797

H -4.219283 3.369494 -2.838004

H 1.658093 4.229140 -3.152455

H 2.284525 -0.642657 -4.731878

H -2.462743 -4.094526 3.073694

H 2.670912 -3.322471 2.893292

H -1.342555 5.462033 1.853402

H 1.851244 2.283332 4.652741

H -3.278423 1.464506 4.783242

Sn 5.089389 -0.523495 0.841161

I 6.735590 -2.482874 -0.253307

I 6.037496 1.728535 -0.745975

H 3.447281 2.757634 1.533648

H 3.911211 2.497371 0.019118

H -12.427720 -0.417294 -0.121701

**FASnI_3_**

Sn I C H N

1.0000000000000000

17.5857999999999990 0.0000000000000000 0.0000000000000000

0.0000000000000000 12.8148000000000000 0.0000000000000000

0.0000000000000000 0.0000000000000000 46.1650999999999954

Sn I C H N

12 36 12 60 24

Selective dynamics

Cartesian

-0.0805700601926326 9.6699240608606001 3.5596711395161664 T T T

0.2864759338788800 9.9714557356680285 13.0585952737013926 T T T

-0.0970729683064506 3.4375636542336272 3.3849261119727774 T T T

-0.0298256586607447 3.4210131718973749 13.7874389078043063 T T T

4.9123637766097161 9.8829946463930636 8.3529776451313644 T T T

4.4775234105997281 3.3298661685951183 8.7859725414032397 T T T

8.7137466194428548 9.6649571286933504 3.5603599038723730 T T T

9.0754496782259793 10.0216163941481451 13.0688081862149730 T T T

8.6981305980778103 3.4093720073696376 3.3891336755900419 T T T

8.7908761451540034 3.4434677652537768 13.7737536812189312 T T T

13.7074480547472177 9.9070470234402990 8.3512729211230532 T T T

13.2635141044608709 3.3625734323535137 8.7381169094669122 T T T

0.0885835381966859 6.9617261245989042 4.7981311628944985 T T T

0.0371936170301552 7.1217444072567151 13.5185953904088034 T T T

0.1695374067204164 0.6879098737891455 4.4210392568614267 T T T

-0.6040150179816887 0.7239838668485913 14.7954154788930605 T T T

5.5950933994589640 6.9918589920832748 8.0716175933465326 T T T

4.4046590272226158 0.4451814155437889 9.3760422126287271 T T T

1.4521474349999999 3.0818312520000002 0.9998610499999999 F F F

2.2102932047512933 2.9346541366930654 11.9493082852840420 T T T

1.9478911369999998 9.2808626040000011 1.4958484609999998 F F F

1.9401464986253354 9.6958027667278071 10.6547704655749378 T T T

6.6588918212792745 3.2065275383257781 6.9112125661141173 T T T

7.0737162178444137 10.5832158638664691 6.6537764710119207 T T T

5.8485974349999994 3.0818312520000002 2.4920480480000000 F F F

6.7797273130384772 3.2906560105188061 11.7058073932204465 T T T

6.3443411369999998 9.2808626040000011 1.9963422879999999 F F F

6.6339630155353664 10.2693384579391935 11.5422944060395931 T T T

2.4250967280509124 3.4518036292219976 6.7581624409884435 T T T

2.8756150977474029 9.9722661818031160 6.2451873466581578 T T T

8.8836039048394611 6.9412444036273824 4.7573170862780056 T T T

8.8585448134533316 7.1901477132119807 13.6534265353435220 T T T

8.9690398448197648 0.6567937801389349 4.4148117602582762 T T T

8.2287051146304879 0.7650861008036529 14.8453621794580553 T T T

14.4009645605451304 7.0240303883182253 8.0410140843093849 T T T

13.2135033229118903 0.4798036958111002 9.3231491392420676 T T T

10.2450474350000000 3.0818312520000002 0.9998610499999999 F F F

10.9523115809225775 2.9022104141590592 11.8521166230885076 T T T

10.7407911369999987 9.2808626040000011 1.4958484609999998 F F F

10.6955904135690538 9.6550206490031805 10.6467267178266791 T T T

15.4823335467681975 3.2441013175583526 6.9154704993649760 T T T

15.8368309157595402 10.6230232821252333 6.6256872316207955 T T T

14.6414974349999980 3.0818312520000002 2.4920480480000000 F F F

15.6354182846888765 3.2983931010054621 11.6286907997317783 T T T

15.1372411370000002 9.2808626040000011 1.9963422879999999 F F F

15.4667156489901281 10.2659893847040671 11.4880518665994806 T T T

11.2477627024490605 3.4596659235856349 6.6662503635349006 T T T

11.6574734168153977 10.0038118069813535 6.2586158898620932 T T T

0.2526914733650309 12.6444527722653817 8.6924419533436215 T T T

0.6712333835710340 6.8000924069176536 8.1535823023815457 T T T

4.1916512000587458 12.0572865233458835 3.2484601500071526 T T T

4.3884357543866086 12.5736361841003035 13.3322020842674931 T T T

4.4603737159375694 6.6662304773810117 3.8600238588190638 T T T

4.4178020589440976 7.1083231432862126 11.6505026401278133 T T T

9.0289964489653887 12.6493464013567181 8.6902061741801617 T T T

9.4791853104664074 6.7970567541397351 8.0744860319985321 T T T

12.9762159889745181 12.0643986073738407 3.2425886710732028 T T T

13.2183417568169101 12.5776772060260065 13.2501847430495800 T T T

13.2558503438056494 6.6720805447301661 3.8569026536553883 T T T

13.2003101528935876 7.0784225087986101 11.5752138297481117 T T T

0.3686856173751393 11.7632806972260457 9.3274136400275101 T T T

0.0354274109088215 7.6887582519995270 8.1248781611268193 T T T

4.1668906349638331 10.9722750259090169 3.3710211775343462 T T T

5.0185838812801356 11.7168171104842571 13.5781847126768103 T T T

4.3240234492071430 7.7246557334401817 4.0917133535660568 T T T

4.3569904365382648 8.1905506746311598 11.5058663714381897 T T T

0.9980210822415349 0.6983714325767720 6.9875693061716975 T T T

1.7938357057981826 12.0614865279858758 7.5215294511590702 T T T

2.5569255290021298 6.1312821260165888 7.7692438324975939 T T T

2.2808368077428041 7.8394763019703175 7.5272756448651448 T T T

7.8090769857917621 1.4651587516300266 8.4062581960439697 T T T

7.3314780435691924 13.1809980062439571 9.6576624901080077 T T T

9.3969670707359096 4.7688166380686452 8.2076925880321721 T T T

7.8780605600175457 5.6277160314712926 8.3847222135134363 T T T

3.2248496291328728 0.7520862328832701 2.2149789621724447 T T T

2.7201128404227699 0.3188652152200836 12.3175268735840309 T T T

2.6871697575616964 11.9159685206790851 1.9361801667496139 T T T

3.0968837290038969 11.4320700541911418 12.2527622153472588 T T T

3.7513292073587028 5.1725777247856470 2.6438659874399093 T T T

3.2252071902167554 5.4136840216053459 11.5999223470025417 T T T

2.9711209099999998 6.7418662800000000 2.5190865439999999 F F F

2.4647341822363296 6.9513665954689996 11.2359027397432829 T T T

5.2577051585126791 0.9186449181990185 3.7782184586013274 T T T

4.3257960681645109 1.7909946166085839 13.3766208556767907 T T T

5.7383459901792389 12.2275303471256596 4.5341204189935151 T T T

5.7492420858590849 13.8515485405972179 14.1070699954484695 T T T

5.6177391148720579 5.0343366654333188 4.2323349610416798 T T T

5.7762506494942309 5.6074971149247697 12.0666362437161858 T T T

6.0760708929813108 6.5004966633728287 5.0747984368482397 T T T

6.3756483600836820 7.2475397095767500 12.0996167955672789 T T T

9.1363762966032471 11.7435627445910313 9.2908126991290665 T T T

8.8485968460280553 7.6891222666043628 8.0485816091821398 T T T

12.9408034007288748 10.9811131494928276 3.3771595840380337 T T T

13.8574975172543251 11.7459090473237993 13.5525457115494596 T T T

13.1045218448778442 7.7255472760119899 4.1013267568793967 T T T

13.1331245034421151 8.1605655144545217 11.4315313960959237 T T T

9.8106562140332443 0.7819968287459215 7.0436378745431174 T T T

10.5824322970133782 12.1109737033605338 7.5163911973749853 T T T

11.3717124546251043 6.1311945645019152 7.7225585741096561 T T T

11.0998684152181628 7.8397400655072582 7.4884200682759872 T T T

16.6188555918328618 1.4553400666431959 8.3656565157649521 T T T

16.1560979294549441 13.2180574281565573 9.6628023088541219 T T T

18.1863820933552951 4.7733205076855567 8.3201719862987460 T T T

16.6681826661610835 5.6259843175790305 8.5136984939832256 T T T

12.0448070055873728 0.7530279374448750 2.1680726933612013 T T T

11.5611225364233725 0.2358725886173287 12.1747463577988242 T T T

11.5050371685605430 11.9170619164668423 1.8935916398153716 T T T

11.9672336557559298 11.3590935721990576 12.2050102320288634 T T T

12.5650539452571426 5.1815726829775430 2.6266580955200278 T T T

12.0116062139994320 5.3798685217647577 11.5310385063058387 T T T

11.7640209099999993 6.7418662800000000 2.5190865439999999 F F F

11.2406687850918274 6.9190959467086088 11.1933229904558207 T T T

14.0307155862485189 0.9271078091365432 3.7884164115138432 T T T

13.1117029239137537 1.7951323978812899 13.1971041243715295 T T T

14.4846078268012342 12.2409741176095359 4.5735303651475379 T T T

14.5421341789348375 13.9133700860971459 13.9780550308119018 T T T

14.4290589307200765 5.0492872250889489 4.2145868181737631 T T T

14.5687837203870831 5.5789462240842527 11.9699090597330784 T T T

14.8787925106336232 6.5139716393313387 5.0649070218912406 T T T

15.1663966171269884 7.2186266227316196 11.9908247962332144 T T T

1.1154098069999552 12.8029013227509179 7.7210699114730090 T T T

1.9586698502364910 6.9470718631422628 7.9244231662100963 T T T

8.0554226512779099 13.4791225935500183 9.0060585097542205 T T T

8.9015039148311672 5.6519112642196525 8.3723878887075394 T T T

3.3238598818751566 12.5677941737215466 2.4038306791685113 T T T

3.2663205961681716 12.3465952706433324 12.6836440207846390 T T T

3.6471537058585053 6.1337781039997390 2.9739014449082193 T T T

3.3080932309645306 6.4271026307906158 11.4698269425104336 T T T

5.0751562333241367 12.7345500754202554 3.9491596242029350 T T T

4.8100848418513644 13.7621090328982500 13.6962706759874653 T T T

5.3991956574977644 6.0042607364825855 4.4944270515384384 T T T

5.5776787674863657 6.6164055201670422 12.0092722446875158 T T T

9.9075088292820865 12.8487633088731972 7.7399154804013550 T T T

10.7723954528948234 6.9455624220261951 7.8774977308684146 T T T

16.8675078313440601 13.4854111022025105 8.9854806940297767 T T T

17.6901665743333716 5.6569427924728197 8.4803935900512641 T T T

12.1365326743432700 12.5699175596649653 2.3668362207585556 T T T

12.1146327134941565 12.2961810216184926 12.5917360318681020 T T T

12.4487797600726964 6.1381040307124035 2.9662228076499342 T T T

12.0879773543155480 6.3957665519311959 11.4161356339094322 T T T

13.8428218870183279 12.7445890108099071 3.9612652162382420 T T T

13.6115948665527942 13.7911417803660630 13.5575342323440875 T T T

14.2046313554685426 6.0160736626622926 4.4824194365044070 T T T

14.3671856132943301 6.5875102280694717 11.9117666900665036 T T T

**MOF1**

C H O Zr

1.0000000000000000

17.5857999999999990 0.0000000000000000 0.0000000000000000

0.0000000000000000 12.8148000000000000 0.0000000000000000

0.0000000000000000 0.0000000000000000 46.1650999999999954

C H O Zr

19 19 34 6

Cartesian

9.0243444693183221 10.2110615646943810 30.6557540962905186

11.1798534867276320 7.2256302398185426 28.3923980235165700

6.8610295432774224 7.2289553357686742 28.3968887681421300

4.5362004592190379 10.5877236694255643 26.1775302805086270

6.6999662582095434 13.7336157790088134 24.1457080153234322

6.7013854973931029 13.5679768843664661 28.4572683221852607

10.2375203907695536 10.3340462344688238 18.1251827492467363

7.8048754172537080 10.5283922670999548 19.4917119282299538

7.8228728979042224 10.4728779071788196 18.1030447040436080

10.2356748844077039 10.3946935251304211 19.5132471310150315

9.0100552523867474 10.5311519656047352 21.6790863685684236

9.0445020104688680 10.2817464901744540 15.8567038419773674

6.8565308709135628 7.3778690309031143 23.7512281281796405

11.1804238140778818 7.3748552785859447 23.7487258558349446

9.0348267760918297 10.3682567289988565 17.3996977147460363

9.0153583343244854 10.4869786192709338 20.2156831141872253

13.5035763035060477 10.5821801228843544 26.1684235739839721

11.3506600206959209 13.5631754093650443 28.4540446249497059

11.3438850397247979 13.7242683777764025 24.1368356422242094

6.2355659121635947 8.5046755869648667 26.1018957998110643

9.0247940779343185 12.3472894980223451 29.0278934076026367

11.8012484412940122 8.5009688972636166 26.0946380222058067

11.1622039740510637 10.2541959095410320 17.5509622229168372

6.8652177046526619 10.6034583244627356 20.0405652086582862

6.9069958657088462 10.5012882093527580 17.5099839405499758

11.1668889456047697 10.3657406978536741 20.0800604856187341

9.0180706535003985 12.5135116681716934 23.4426183661805219

6.3814504119636482 6.6156538695409628 23.1033898532110911

3.4306871419770082 10.6739863063874907 26.1828729277914292

11.6571657206915837 6.6120214340121635 23.1028036261068159

9.0247913712592194 10.0817471100386040 31.7589953306384167

11.6665205590616949 6.4213330829324873 28.9818773494765871

6.3736272019477234 6.4253771417275809 28.9867512044272786

6.1031580977906952 14.5375561166679432 23.6715387676699756

6.0821160275451360 14.3322925181085292 28.9704816761872266

14.6091450164443923 10.6681717239909961 26.1719655154706281

11.9717215294828634 14.3261215186212674 28.9672133445126541

11.9446727702373909 14.5252479008652706 23.6627920583418145

10.1675843216422788 10.2790428063371060 30.1079181995437217

10.3667099245952414 6.8416019164509381 27.4982200975197557

6.5287366374149975 8.4187386194875486 28.6980587439668007

7.6727018992609999 6.8436552531763759 27.5019268058854536

11.5142454278655748 8.4149270783982946 28.6931958779355654

7.8804461571026847 10.2815558122541937 30.1093245829154021

9.0208915240267409 9.3773562285665690 27.6202456439836617

7.0259860685336717 9.0655566458749739 26.1301186600485735

9.0238840113304075 11.8142483614433278 28.2185469935822901

5.0877695140773689 10.5759745679460782 25.0363443879204368

7.5847557566263450 14.1131448082263926 24.9769885381070225

6.4392013596487629 12.3574868217276332 28.7451910501958956

7.5873081092982941 14.0124311368750121 27.6662785904448434

6.4258227787134246 12.5472478723680521 23.7894618663840021

5.0912047810943726 10.5028132507604486 27.3199557074105037

7.5511097235544051 11.5159497847072334 26.2138485569624144

11.0116117294469422 9.0627928020819333 26.1254774656339599

10.1889154871471348 10.1938084899972949 15.3246111404836807

7.8776543151644587 10.5628537160225449 22.3049974814628804

7.6721922926716637 6.9388236390956006 24.6238920781746309

11.5209796774891569 8.5792491399491801 23.5393822512117232

10.3639092510658699 6.9372021683108631 24.6210080283136783

6.5156861733130533 8.5823245151407974 23.5435439943309319

7.9070253702570552 10.3044568362412008 15.3037343841906104

10.1410776197377022 10.5449514410774814 22.3081203197766129

9.0161598685761568 9.4846177353675891 24.6815905426583768

12.9510311627237087 10.4965297207995221 27.3121597589002398

10.4655417719299972 14.0093151650574725 27.6630778259556962

11.6085251313977604 12.5370841739916994 23.7755725998422278

10.4651759262512627 14.1069202819004342 24.9728153252158549

11.6102463333942225 12.3521382690704478 28.7419959964108216

12.9496523328815911 10.5713763617147123 25.0285490251760301

10.4909259012536076 11.5126792066955748 26.2103372406102260

9.0182778205069862 11.9660239071516550 24.2441368443923118

7.2528882860835688 10.4160905028686948 27.9306482302793668

9.0185323760477214 7.9664137275877591 26.0793682170585406

10.7917961993993430 10.4131153503132285 27.9272517429013263

7.2502594906892384 10.5378363689489092 24.3757362672711189

9.0233103913063140 12.9823015399183408 26.2563914836121413

10.7837928955567506 10.5330701318348119 24.3711964139090433

**MOF2**

C H O Zr H

1.0000000000000000

17.5857999999999990 0.0000000000000000 0.0000000000000000

0.0000000000000000 12.8148000000000000 0.0000000000000000

0.0000000000000000 0.0000000000000000 46.1650999999999954

C H O Zr H

19 19 34 6 1

Cartesian

9.0251724246192708 10.2006844744643441 30.6662561042072461

11.1801040719081239 7.2227414218570933 28.3932464929586992

6.8577652630971233 7.2274009091901652 28.4006065463420363

4.5353287427719069 10.5939661973062442 26.1997362971631382

6.7005773666450050 13.7240970383272884 24.1459764707314015

6.7016364241162174 13.5684859296174949 28.4632772593897911

10.2508043403755238 10.3794772893909002 18.1200997826765615

7.8092020625133953 10.4812788177778291 19.4910732743945054

7.8214002080562626 10.4682754589856941 18.1011091576928571

10.2374638296233513 10.3845232859967886 19.5115676844539188

9.0105406589057040 10.4986959168115757 21.6975275845139670

8.9979964561444259 10.4517295647365227 15.9227252613951062

6.8540245891160199 7.3913652283332461 23.7552567583224423

11.1737052726474158 7.3868150444835265 23.7499240239991352

9.0415495487678346 10.4298810423013464 17.4090026471893005

9.0182155831187298 10.4468528567874532 20.2033782285139445

13.5008946679109680 10.5853031528480397 26.1835441193051679

11.3510141181174546 13.5615298727283342 28.4577391953824410

11.3400343473297660 13.7142215545772839 24.1342600875329332

6.2277578999404613 8.5101504969538109 26.1248302505824093

9.0255140615789511 12.3454967136658578 29.0447269236728189

11.8027763855581789 8.5029779725314878 26.1138621754329634

11.1931097884439197 10.3383002833293638 17.5757268568846108

6.8718848585339476 10.5267582004147133 20.0443953748659602

6.8971540750455080 10.4963923436023521 17.5239495492913449

11.1656348735023077 10.3482967612740691 20.0808316476938202

9.0142908726217463 12.5347157938924099 23.4698047490542798

6.3743579556995913 6.6327234590588882 23.1052732108411938

3.4304336842284537 10.6792924856177649 26.2053618901673993

11.6542990076479267 6.6268765196723702 23.1020442262409773

9.0265935131664090 10.0727658619572740 31.7667698363442916

11.6699472199441381 6.4189908255218704 28.9776997435248198

6.3683483580097766 6.4245065338782323 28.9867535833251253

6.0895792569033391 14.5256247320289980 23.6856477331728286

6.0850822046686428 14.3325855585898392 28.9772317713874372

14.6057765137426649 10.6707092827907051 26.1853262893126839

11.9690734705484001 14.3236993592323287 28.9727328455845132

11.9537818205485600 14.5124064241968345 23.6716273758409450

10.1655974020631934 10.2678260378838928 30.1106201791243215

10.3640630345611733 6.8429420135479493 27.4974360272379776

6.5318270945547434 8.4178932775135387 28.7017758122690054

7.6699727211579241 6.8453703222808668 27.5020749449693476

11.5098078273812305 8.4124789483179807 28.6937944563978675

7.8835563598195577 10.2722691735666150 30.1137123105220716

9.0193904788737260 9.3836272626625465 27.6223869797525694

7.0208983192401844 9.0681655487109651 26.1434040019897083

9.0240196035121212 11.8138891609559433 28.2334970710362647

5.0891441343686257 10.5881198000851775 25.0563075228153700

7.5845989972778085 14.1041573681932100 24.9747137614537742

6.4433094794330161 12.3577802313285794 28.7496854772035242

7.5867030291599544 14.0093098964521854 27.6664528073179632

6.4383295622471115 12.5374128254665234 23.7742355789062678

5.0933932757349760 10.5039047201653997 27.3372334162917880

7.5493399495131071 11.5169596286483529 26.2293348896286744

11.0115744244702984 9.0634930168711101 26.1365890012879625

10.2385072761435687 10.5582430616343537 15.3566338352234855

7.8738533531123123 10.5355628930784224 22.2813496289237420

7.6678239100137100 6.9481539280357172 24.6236685036563188

11.5121767945471980 8.5934255501674546 23.5388374253775901

10.3591511597765766 6.9460666721107032 24.6186067349216842

6.5162200435674986 8.5985419752883239 23.5467916014044754

7.9782396624463869 10.3918045724637160 15.2507246478150318

10.1450849280987363 10.5246156712822589 22.2861764967708069

9.0133846537512667 9.4856298877748717 24.6880526920954573

12.9469277860857979 10.4936675634675982 27.3230522950262973

10.4668802594614530 14.0048867654822988 27.6612759258173604

11.5932125825221917 12.5265908690374559 23.7592871418034868

10.4629001651471629 14.0979231142996451 24.9684406215498100

11.6068384630288008 12.3500018578045125 28.7430481303283081

12.9430503603042766 10.5811462077382696 25.0422876895148505

10.4897030821212258 11.5126096435947769 26.2247462080305347

9.0158844052840124 11.9538252142493100 24.2465900085236150

7.2493414304021879 10.4149817983665010 27.9644951231533625

9.0151600456880754 7.9658806536341578 26.1018493939284362

10.7940044686955883 10.4094678060655355 27.9594943894504304

7.2435934827863884 10.5391052803385268 24.4214709816731279

9.0227737102164962 12.9824929862019776 26.2783266401954556

10.7857248395435281 10.5339351819518541 24.4155640971716394

10.0875847760767776 10.5645815839358850 14.3883867979917195

**MOF3**

C H O Zr H

1.0000000000000000

17.5857999999999990 0.0000000000000000 0.0000000000000000

0.0000000000000000 12.8148000000000000 0.0000000000000000

0.0000000000000000 0.0000000000000000 46.1650999999999954

C H O Zr H

18 21 34 6 1

Cartesian

11.0251413815876482 13.7401698543427830 16.9374665935519957

6.7270762378536038 13.7136768266989435 16.9388317382100375

4.3974081415399295 10.3557168465249063 19.1725800577167824

6.5816422920146396 7.1833316369633158 21.1965559126863390

6.5219644237384662 7.3606741890147447 16.9045148538423966

10.1820650160161410 10.5841707335574533 27.1831500422883501

7.7273358222316162 10.3697033920596500 25.8473625056043410

7.7540327476672326 10.4648877913928029 27.2340904476180476

10.1551660492351807 10.4764870924039908 25.7971169075742885

8.9099758340305470 10.3446503470388063 23.6283205381007519

8.9469309844637017 10.7523488370109863 29.3849463943496829

6.7534185154457225 13.5126770027335059 21.6081526034569222

11.0687850248661217 13.4987489572558186 21.5807098286369694

8.9794429918556542 10.5903254148675678 27.9072536752662259

8.9291121224010954 10.3870700308138666 25.1220015505424143

13.3810431452689880 10.3547434751526115 19.1012679157620191

11.2261799179377828 7.3084581692759469 16.9334978626698884

11.2163541134850941 7.1844763137577319 21.1750352922486513

10.6145094663625876 11.2747608947484359 14.7815826096982637

6.1003035416945623 12.4165152027590526 19.2465725045356741

8.8857143897095963 8.6130932671463505 16.2251992307353206

11.6733623051778217 12.4119779469892091 19.2126692252489519

11.1316361744263599 10.6743359979057733 27.7077376173304515

6.7846444679668139 10.2937097211686535 25.3060194484742347

6.8348940847871580 10.4655300906143918 27.8199824909697391

11.0785959988630456 10.4747848209660166 25.2190173407816047

8.9017108818458350 8.3617325681079375 21.8454974068597814

6.2774012019993135 14.2693743626560980 22.2626961430864618

3.2919520629021624 10.2808639721273334 19.1773267909965597

11.5621978841558875 14.2520203051495145 22.2263680690222962

11.5016671977344878 14.5630098825179743 16.3671898221580463

6.2377451520241367 14.5234082551596337 16.3607282298146544

5.9781564838660630 6.3835405514180055 21.6699110903528336

5.8959148723639592 6.5995544065711220 16.3972697157559040

14.4867335683850893 10.2827852401599245 19.0965178446437278

11.8507624519465171 6.5299553320869865 16.4511335948908872

11.8282395753084373 6.3863949125529835 21.6403708525977407

8.7470448985942735 10.6432051767695679 15.1310203624647972

7.3209419667878768 11.4503052352707151 14.8236525750186541

10.1666057455710170 10.5603002475928900 15.2660097087980393

10.2177826836333150 14.0928018679919269 17.8515760537113941

6.4171830710370852 12.5230910033552032 16.6003329158746809

7.5188625520868841 14.0802153048586582 17.8554029853057230

11.3569324099814271 12.5604366586618426 16.6004000921458861

7.6679345588990913 10.6240238648821048 15.2045081339704886

8.8777000615397501 11.5365648547089528 17.7159219744160907

6.8894726058629363 11.8544539553115449 19.2055928804735068

8.8754971008341190 9.1268943033996521 17.0497934954914285

4.9649802228701452 10.3344637261722028 20.3082918265781842

7.4572692526761841 6.8022879950211363 20.3609525373363560

6.2512012227777474 8.5728536542463889 16.6418198514336702

7.4288596700453411 6.9145299478224684 17.6725047003438291

6.3179591141049034 8.3713482242759110 21.5656767199996615

4.9434256909650180 10.4625106623339938 18.0298033772864592

7.4137918955643922 9.3950629947376800 19.1098293846592462

10.8836587328408072 11.8503532540000442 19.1765010354386192

10.1760572072362283 11.0294547572645918 29.9174903354732820

7.7665164040947667 10.3256223961036255 23.0549877917987338

7.5564171115609495 13.9595585721427451 20.7318986939177883

11.3991858091105751 12.2884951203772843 21.7820077192539792

10.2500024904206484 13.9506124722195270 20.7216929237549934

6.4214653078906121 12.3040220203162463 21.8182198231577544

7.9419476332809804 10.6709689410793302 30.0767322341145480

10.0383942137780835 10.3333879540377378 23.0296172453950980

8.8942143531279196 11.4188009001848449 20.6520624011629792

12.8264648903204641 10.4719031364603090 17.9641439153028202

10.2780031801367535 6.8782090941923615 17.6681014998412600

11.4831114151728499 8.3732680448379870 21.5389046830575559

10.3294923915810948 6.8015981822545690 20.3521884641017756

11.5295686894699863 8.5101723179325344 16.6854262157797351

12.8232226282873718 10.3209183412353305 20.2415610891567077

10.3454785492066073 9.3895752781285324 19.0874751409322343

8.8951824351376381 8.9457654356084326 21.0714878003280894

7.1011453563503988 10.5052016400755726 17.4030571207341573

8.8927443911965103 12.9386921562383428 19.2432989478362266

10.6718775742617140 10.5089160475274372 17.3398947129926597

7.1278985753776514 10.3645356510773770 20.9267299833656466

8.8888880353786082 7.9255474838981144 19.0376607187399998

10.6673268764568085 10.3577388597348676 20.8875044533308234

10.0281896285314218 11.1314145011690684 30.8811600729097684

**B1**

Sn I C H N O Zr

1.0000000000000000

17.5857999999999990 0.0000000000000000 0.0000000000000000

0.0000000000000000 12.8148000000000000 0.0000000000000000

0.0000000000000000 0.0000000000000000 46.1650999999999954

Sn I C H N O Zr

12 36 31 79 24 34 6

Selective dynamics

Cartesian

-0.0862252905257827 9.6217995509733498 3.5926305818281747 T T T

0.2596569411265901 10.0750906567426615 12.9854801452546003 T T T

-0.0963720468134091 3.4150909618882999 3.4061053900110929 T T T

-0.0832148604151531 3.4241566424955825 13.7660714034942515 T T T

4.8761158415099342 9.9080392448437031 8.3680018745840865 T T T

4.4248394902989583 3.3268429785141183 8.7735275055206312 T T T

8.7096676839060851 9.5990793290469885 3.5912987724172694 T T T

9.0219650815688670 10.1816546703980002 13.1671916200993806 T T T

8.6991738334127522 3.3997163581628427 3.4117345677777839 T T T

8.7989391786507980 3.7121661959443197 13.6794329446764724 T T T

13.6627740035527534 9.9538060971949918 8.3437071756840488 T T T

13.2560454273553141 3.3723106217322201 8.7072966039433819 T T T

0.1034512646880317 6.8835983565010768 4.7206138442068806 T T T

0.0020604482540053 7.2259277351902043 13.5767890671461000 T T T

0.1943388804472626 0.6600887982243966 4.3571263194489758 T T T

-0.7190930731976083 0.7745443985303182 14.8808382244521731 T T T

5.6127022239528870 6.9968676843633144 8.1158586083185043 T T T

4.2932797193999805 0.4606263312591958 9.3733357284382866 T T T

1.4521474349999999 3.0818312520000002 0.9998610499999999 F F F

2.2215625396499346 2.8727051100235448 12.0353062336428120 T T T

1.9478911369999998 9.2808626040000011 1.4958484609999998 F F F

1.9313291875107956 9.6788748225254828 10.6247697207711465 T T T

6.6720838750328175 3.0893471408365230 6.9818236616537552 T T T

7.0157132081099549 10.5780404323882458 6.6097056462332802 T T T

5.8485974349999994 3.0818312520000002 2.4920480480000000 F F F

6.7444593232983765 3.3260901005478956 11.6580392119248639 T T T

6.3443411369999998 9.2808626040000011 1.9963422879999999 F F F

6.4634519239365211 10.2564064775429440 11.4392394356887337 T T T

2.4211335695295513 3.4516697418522186 6.6689632170930961 T T T

2.8100308726879160 9.9505196506134848 6.2175982318723788 T T T

8.8808221634384275 6.8590200564862540 4.7004547560435448 T T T

8.9045005618323803 7.2705281136098590 13.4610371879778405 T T T

8.9735253333574860 0.6424482917033489 4.4091992227429717 T T T

8.3339028235498294 1.1540591717018698 15.0518751397662172 T T T

14.4420851831875652 7.0500849966076862 8.1083846649433049 T T T

13.1833044738211242 0.4935308904793657 9.2689472639014241 T T T

10.2450474350000000 3.0818312520000002 0.9998610499999999 F F F

10.9652517107858802 2.9980781223753938 11.8026877486779771 T T T

10.7407911369999987 9.2808626040000011 1.4958484609999998 F F F

10.8652564989352616 9.5909597219674687 10.4231872936506775 T T T

15.5062326930677337 3.2180333518713269 6.9035383861803048 T T T

15.8435538742633142 10.6075221207292678 6.5648294284561901 T T T

14.6414974349999980 3.0818312520000002 2.4920480480000000 F F F

15.6221839110903709 3.2112116140729539 11.5945566602275445 T T T

15.1372411370000002 9.2808626040000011 1.9963422879999999 F F F

15.4434554778465785 10.3348816276814581 11.4180137037304199 T T T

11.2314275352640891 3.4248372580137167 6.6333161306198187 T T T

11.6426473346616373 9.9675609186521310 6.2137197488422844 T T T

0.2081500586760938 12.5820361988442819 8.5972633563149845 T T T

0.6899725354231756 6.7787978808464331 8.1126694548959701 T T T

4.2548700249571771 12.0438504670311168 3.3733727894954106 T T T

4.5638985233637204 12.5088690619711365 13.3929270449419100 T T T

4.3558900870638659 6.6110820941080410 3.9673019259568436 T T T

4.4050986284330884 7.1225529686584812 11.7095454527380518 T T T

8.9726843798315734 12.5079602013574682 8.7786614353484911 T T T

9.4992190458180303 6.7448556225652094 8.1450560333923416 T T T

13.0450334274078728 12.0550918122604660 3.3571488097870170 T T T

12.9623061621774376 12.4549370450324339 13.1378217185206712 T T T

13.1562023394818031 6.6453759624352564 3.9623520643195485 T T T

13.2188804367129560 7.0846352933365093 11.6622716033285325 T T T

9.0275121610183771 10.2013652810026390 30.6580363733673202 T T T

11.1820666821757033 7.2300035378425793 28.3786283790353053 T T T

6.8537206334585932 7.2374285512449728 28.3894473161495604 T T T

4.5355078822767396 10.6119185683668587 26.1961956783630896 T T T

6.6980485938486982 13.7470305501407122 24.1549044705899938 T T T

6.7039278402777311 13.5774362920358040 28.4717645621480813 T T T

10.2544281251617146 10.3696897335666378 18.1325196745931478 T T T

7.8094357496369673 10.5091893872529862 19.4845291587454419 T T T

7.8301582755717911 10.4551307428342231 18.0943710071779513 T T T

10.2372562738656629 10.4266003094563793 19.5206527601722541 T T T

9.0038659687334288 10.5496168388454326 21.6927705394728569 T T T

9.0909430537790890 10.2903087102029343 15.9146909615835987 T T T

6.8472677781579305 7.4135945189689716 23.7480035900222859 T T T

11.1634098186909654 7.4057494805862056 23.7351185512643887 T T T

9.0520189649521896 10.3834279730850785 17.4102887010784428 T T T

9.0139098800881055 10.4955109433109897 20.2078387436897131 T T T

13.5000388581563247 10.5972259796967165 26.1758258638302834 T T T

11.3532676951054246 13.5687543469478769 28.4606424638142919 T T T

11.3350121867244962 13.7383655456682821 24.1399445901815000 T T T

0.2740351420220734 11.6264868291644561 9.1210953849447822 T T T

0.0577857992327077 7.6705625809645595 8.0933200910256460 T T T

4.2578884896255760 10.9630696310906011 3.5277947490629731 T T T

5.1521412088790548 11.6582142256488446 13.7416406290482538 T T T

4.1547839895722394 7.6405201816763109 4.2730566582279241 T T T

4.3001660454069155 8.2042668789092570 11.5809245738485984 T T T

1.0574425052296976 0.8247599861309854 7.0540346110300787 T T T

1.7369300003756114 12.0702856044183768 7.3833810869842758 T T T

2.5726527663899201 6.1076350460157105 7.7148857352038869 T T T

2.2950353039448625 7.8183293583114324 7.4751247998758243 T T T

7.6377043622847918 1.2460540486662031 8.5928856674832250 T T T

7.3071306318497271 12.8999661288194929 9.8494665971430191 T T T

9.3516821872899936 4.7129624518484654 8.2037089068657956 T T T

7.8825779931824682 5.6155154991528695 8.5319692001646033 T T T

3.2567896119031490 0.6867013198479969 2.3021648650950479 T T T

2.9848423202567438 0.2120572932869781 12.2209342808950669 T T T

2.7667806640883481 11.8310329742892684 2.0466600718453845 T T T

3.3334703744539489 11.3269227299514057 12.2939099053693273 T T T

3.7907627319012174 5.1881749995020243 2.6013827704441219 T T T

3.2802337977046050 5.3797445801324582 11.6592551689518498 T T T

2.9711209099999998 6.7418662800000000 2.5190865439999999 F F F

2.4494599362850815 6.8876514808239229 11.3361035645088695 T T T

5.2712946439197754 0.9446733730492305 3.8717000578531522 T T T

4.5431562994442540 1.7290798085013699 13.2994605629775258 T T T

5.7723726678587086 12.2862451032526110 4.6829071667418436 T T T

5.8948865341775996 13.8290882373496409 14.1713019662115407 T T T

5.5323029261293639 4.9861342199104675 4.3310034925256078 T T T

5.8290140224325055 5.6739792917880481 12.0644143813455873 T T T

5.8006430053536393 6.3802981027350469 5.3692828793856719 T T T

6.3690884664690834 7.3398324997030331 12.1042215479212629 T T T

9.1799410046203924 11.6047787612944262 9.3611215671655206 T T T

8.9075649426485253 7.6597237370676243 8.2258810668113753 T T T

13.0210749397286598 10.9760684714917787 3.5212319175183540 T T T

13.4790756629818684 11.5547523083143044 13.4719823730978288 T T T

12.9331791911313374 7.6701425373569476 4.2685235880315000 T T T

13.2176236954059370 8.1722548967162343 11.5420862763766863 T T T

9.6081486807864618 0.6994852498307379 7.0962918259401384 T T T

10.4965416362809307 12.0851986855782290 7.5296924532805187 T T T

11.3218894052738221 6.0289851047850265 7.5885196633546190 T T T

11.1175387549775433 7.7691127631379322 7.5153788066744109 T T T

16.6378545392100214 1.4589633077176829 8.4419083248274784 T T T

16.1217336816275676 13.1014935408148805 9.6069170827095434 T T T

18.1912203761728115 4.7514865466131786 8.2741088954964539 T T T

16.6830118493047159 5.6196490664702621 8.4928060443157634 T T T

12.0951891394593716 0.7119490370106496 2.2607711911542925 T T T

11.4253414154986341 0.3096419987921381 11.9997213681560915 T T T

11.5684762963047660 11.8673215821204767 2.0126110037939293 T T T

11.6301869280618781 11.3851527632241627 12.0189735471666150 T T T

12.6212502961818185 5.2074067873385665 2.6021158481708682 T T T

11.9326395490644632 5.4674582883747087 11.5416521425521967 T T T

11.7640209099999993 6.7418662800000000 2.5190865439999999 F F F

11.2817781375763104 7.0730862055137500 11.1881204469532136 T T T

14.0847602423844123 0.9394102025071045 3.8579469649750062 T T T

13.0978835472861235 1.6703616692253007 13.0882139728462494 T T T

14.5500977987538214 12.2756767479574584 4.6858460190134572 T T T

14.3920528275396737 13.6538454093823542 13.9434653553652996 T T T

14.3635738698219964 5.0443793888992090 4.3291929257124151 T T T

14.4814871553629079 5.4964979808050280 12.0533016124559502 T T T

14.6091913795176680 6.4450661717541484 5.3623039555151806 T T T

15.1790659027886807 7.0855569691561842 12.1622151739307167 T T T

6.2285269969313726 8.5251921066546359 26.1149221963302480 T T T

9.0269533617374940 12.3494093494910224 29.0482608040726227 T T T

11.7993681818248586 8.5149717086576135 26.1006705531380661 T T T

11.1928735285114378 10.3081440512284512 17.5821223009286989 T T T

6.8660715894864763 10.5588908098099949 20.0269388346735440 T T T

6.9061314552469417 10.4619741596092357 17.5165713307235151 T T T

11.1647243892191099 10.4119308416825884 20.0912938488894106 T T T

9.0134122401602674 12.5518718417687172 23.4692554301094169 T T T

6.3649043093604840 6.6557426100812496 23.0997856062090712 T T T

3.4308764284714584 10.7027570147180864 26.2032965329123435 T T T

11.6359875482540520 6.6463130723160893 23.0818165638094506 T T T

9.0296897943749617 10.0704978372576015 31.7577746940798349 T T T

11.6732262844461321 6.4243433419278029 28.9599000027978164 T T T

6.3638761729482587 6.4335692614427407 28.9745270545236160 T T T

6.0933149212680542 14.5507684435403828 23.6906187805102952 T T T

6.0915079305316073 14.3391716700711420 28.9941899849432190 T T T

14.6050933024412348 10.6845595111601881 26.1778089706736878 T T T

11.9715051167705742 14.3280537031746906 28.9796529482525038 T T T

11.9376829798415240 14.5397970973825164 23.6690934559225070 T T T

1.1013885372644856 12.8222235429459595 7.6711428507885042 T T T

1.9734484999982711 6.9224439613580264 7.8669177306109379 T T T

7.9578155080071147 13.2597013609731107 9.1535078169049395 T T T

8.8993475059527896 5.6057414483493782 8.4242941763864945 T T T

3.3857761869863436 12.5091705279725236 2.5033248616515364 T T T

3.4785055918179659 12.2601368005405025 12.6888763250371284 T T T

3.6481270349236570 6.1302537787171332 2.9690716905787107 T T T

3.3182365871241717 6.3971480996676018 11.5462597698959666 T T T

5.1113421877528706 12.7620843639514021 4.0665069937192690 T T T

4.9852383409068972 13.7139812844414415 13.7027800444152774 T T T

5.2644199578923567 5.9322932273531350 4.6267058386360720 T T T

5.5936703690159559 6.6754639538410707 12.0239139043673955 T T T

9.7857604303517363 12.7758779689935338 7.7864338417477246 T T T

10.7683392488711416 6.8560534943807339 7.8279818643905781 T T T

16.8585919341040231 13.4276328578720889 8.9844440232039950 T T T

17.7050566165538541 5.6387674737376541 8.4427827694692983 T T T

12.1954359713929659 12.5335864977569074 2.4735952118741120 T T T

11.8733877931375318 12.3052864877182166 12.4210231843330838 T T T

12.4548219500694817 6.1460917988826749 2.9688958880253100 T T T

12.0793408963389712 6.4769580475020359 11.4361774011713742 T T T

13.9091132782559033 12.7594332654999292 4.0532409079021532 T T T

13.4799614101074106 13.6177730931701362 13.4740099843507224 T T T

14.0827297945786469 5.9878910384754827 4.6181252631162364 T T T

14.3424669019043396 6.5141308878522057 12.0320211435106792 T T T

10.1677821770981645 10.2707186803709387 30.1018509056264172 T T T

10.3627448993897815 6.8523134640241556 27.4851045636578917 T T T

6.5277374711957288 8.4273864051960121 28.6928599320287780 T T T

7.6672883276506774 6.8564419931943252 27.4919970044339195 T T T

11.5130893457927588 8.4187095062201909 28.6815759134202111 T T T

7.8852026723141071 10.2755051464916232 30.1068338720250921 T T T

9.0189359889698189 9.3916915839769359 27.6166085047530068 T T T

7.0209157458637552 9.0838558111649288 26.1353116499452192 T T T

9.0247999641586336 11.8232520158234991 28.2335548777776175 T T T

5.0874412495669015 10.6044806255403383 25.0527906574005144 T T T

7.5816527139920513 14.1252238024128953 24.9854103970361585 T T T

6.4436491937680112 12.3657651455946365 28.7522179232318464 T T T

7.5877037217115166 14.0216754497282459 27.6758081695400939 T T T

6.4310396792528888 12.5613483833531347 23.7865659119697384 T T T

5.0924627959076716 10.5193980347096367 27.3345141106907583 T T T

7.5495818554055409 11.5325397918109189 26.2293554569553216 T T T

11.0091383784248347 9.0764806024045654 26.1257185678258956 T T T

10.2178210789638761 10.2057726624541676 15.3315468314269943 T T T

7.8696159995337105 10.5829546041817011 22.2907892133212471 T T T

7.6594828258824768 6.9673298271016346 24.6174391037628020 T T T

11.5018481155586745 8.6117445113788662 23.5263203911292607 T T T

10.3541348926859005 6.9626892333407175 24.6089112007869488 T T T

6.5158125448267921 8.6213570030807656 23.5382473804353332 T T T

7.9781235185627688 10.2789920605969947 15.2645393551250095 T T T

10.1363282055381116 10.5715690128838187 22.2958679385639300 T T T

9.0117428243038677 9.5038594415501230 24.6829425553063793 T T T

12.9479138331457211 10.5047665486921264 27.3165791998986833 T T T

10.4677472896799859 14.0165290267760643 27.6685184738715613 T T T

11.5967581008977465 12.5514617580760124 23.7719667252326481 T T T

10.4585183425540666 14.1199286214558786 24.9765725305030131 T T T

11.6098486110367975 12.3562270424015104 28.7409327153594134 T T T

12.9426232943455961 10.5925808198914861 25.0351912111157766 T T T

10.4880034929641077 11.5263866392972876 26.2225462260867523 T T T

9.0146008428535715 11.9816341144747671 24.2540583824843488 T T T

7.2506336800324140 10.4256158815855660 27.9574630738055809 T T T

9.0132660917718432 7.9804188214901304 26.0891543673820507 T T T

10.7928114829544270 10.4186694971711464 27.9493069041476332 T T T

7.2461053025554119 10.5611875412506659 24.4108336996977080 T T T

9.0221872466806108 12.9972964904999735 26.2794797578990220 T T T

10.7805936780523570 10.5538999244887268 24.4022212042156212 T T T

**B2**

Sn I C H N O Zr

1.0000000000000000

17.5857999999999990 0.0000000000000000 0.0000000000000000

0.0000000000000000 12.8148000000000000 0.0000000000000000

0.0000000000000000 0.0000000000000000 46.1650999999999954

Sn I C H N O Zr

12 36 31 80 24 34 6

Selective dynamics

Cartesian

17.5021825195474854 9.7904623094710654 3.5474780692625179 T T T

0.1750746540913455 9.7480828175452139 13.1715013530876277 T T T

17.4944719655217682 3.5651475477842256 3.3661008895171260 T T T

0.1011661907159770 3.4832738135410954 14.1488295281288039 T T T

4.9528747283442041 10.0282174738615701 8.4848395739283795 T T T

4.5746208985321291 3.6165037545042336 9.2888485086935937 T T T

8.7242307264574368 9.7036554194601088 3.5469381626716632 T T T

9.0945157709761411 9.6592280867276301 13.2045011127042020 T T T

8.6942595530855371 3.4745660174031232 3.3904160136868251 T T T

9.0843386814497915 3.7061215267685728 14.1323665535916856 T T T

13.5382677141663006 10.2514309762923883 8.2373567347373271 T T T

13.3682245897460508 3.7998409102067749 9.0116487650190891 T T T

0.1821868727871531 7.1384722000518055 4.8605988302189829 T T T

17.5443813247101588 6.9175704146861889 12.4779018455771791 T T T

0.3656726612553681 0.9214890254664746 4.5364376311030039 T T T

0.0183674229859936 0.5305560532077170 14.6111575569047485 T T T

5.2563008152876067 7.1216169405553496 8.0134801827742681 T T T

4.4177991488707091 0.7053584981716680 10.0479686543484679 T T T

1.4521474349999735 3.0818312520000153 0.9998610499998607 F F F

2.5117254863953331 3.4494547669575617 12.5518228842806217 T T T

1.9478911370000120 9.2808626039999815 1.4958484610000156 F F F

2.2997017189830502 10.2551556720396135 11.2722204963270389 T T T

6.2931419204357457 3.2154793964391719 7.0403518107786605 T T T

7.0904535665047046 10.6192448702819870 6.6644586510449120 T T T

5.8485974349999736 3.0818312520000153 2.4920480480000506 F F F

7.6932129528832718 3.0337216199782295 11.6927718554994726 T T T

6.3443411370000122 9.2808626039999815 1.9963422880000075 F F F

6.4985826215708338 10.3754171446809522 12.1211363035772148 T T T

2.1098801305285559 3.7944894943696168 7.8078289514151260 T T T

2.8625052207575701 10.2715661527134916 6.4692295241908431 T T T

8.9483246843429587 6.9787586168414704 4.6970242195664529 T T T

8.7238113425480304 6.8632027577152250 12.4061313266125470 T T T

8.9756824552034509 0.7693161143436045 4.5677892469974850 T T T

9.1345732573452683 0.8784236112488506 15.1468287773550774 T T T

13.9153356929136027 7.3299684150624689 7.7101210166005618 T T T

13.1176192402108480 0.9232666398839594 9.6992829958916200 T T T

10.2450474349999734 3.0818312520000153 0.9998610499998607 F F F

11.7434902285944123 3.6215800206810389 12.9618722660968011 T T T

10.7407911370000111 9.2808626039999815 1.4958484610000156 F F F

10.5548624294490860 10.2938840364742248 10.6597912869417897 T T T

15.5001345088572382 3.4090105102601811 7.1245388477700065 T T T

15.7632759685281307 10.9079626200714763 6.5632949040721247 T T T

14.6414974349999731 3.0818312520000153 2.4920480480000506 F F F

15.8648264572865951 3.2759549688911398 11.9520758434178891 T T T

15.1372411370000108 9.2808626039999815 1.9963422880000075 F F F

15.6758113371808125 10.4342046407665450 11.2830164796667169 T T T

11.3036009926255634 3.9019284330199624 7.0048251256818661 T T T

11.4409082119394387 10.4493368529625847 6.2623185496502307 T T T

0.3073080208370380 0.0575593950210872 8.9655348537227706 T T T

0.2725551927753783 7.3825857363318681 8.3044385195630035 T T T

4.2330890998404209 12.0530267339946828 3.2809152585649555 T T T

4.5765636452858063 0.3884699368556318 14.2619913347742830 T T T

4.4988591503785109 6.6992382947810336 3.8209977957390509 T T T

4.5400352096719390 6.9265452334483362 11.6704211219241358 T T T

9.1335070715687134 0.6054264180937733 9.0139080173935202 T T T

9.0733245081335046 7.1285469120067360 8.1305075692587501 T T T

12.9346691528585698 12.0955425102886718 3.2288593330035074 T T T

13.2604339977763122 12.6939496724736287 13.2414929436426885 T T T

13.3427485828694579 6.7082973758896962 3.7629899320412159 T T T

13.2133374478910923 7.2726766189919037 11.2861765960120550 T T T

8.2809660891634103 10.0322177627004567 30.7762448794230359 T T T

10.1696685804141502 7.1685331617925385 28.1560273639767615 T T T

5.8733449730841389 7.2426150639798657 28.5311690769014454 T T T

3.4425717695204008 10.7806403957909129 26.7407530716810911 T T T

5.4924928348045299 1.1779895740323671 24.7018340228103277 T T T

5.8592752768521574 0.7538780211951264 28.9925678609628363 T T T

8.3402482123821322 10.0364180586896747 18.2572488997366662 T T T

6.1013889062494471 10.7526418184801038 19.7742606526272304 T T T

5.9705042253339267 10.5594649584266378 18.4018432185348253 T T T

8.4757788169235670 10.2673138814309528 19.6209239169785903 T T T

7.4981841570394492 10.8126453353866765 21.8714293536689333 T T T

7.0008812481785450 9.9354995495189247 16.1809643453355960 T T T

5.4746881891689547 7.6927019890864567 23.9248213052477006 T T T

9.7902002512800443 7.6228272085566617 23.5491484706418461 T T T

7.0895400828114532 10.1907183092930289 17.6370878892197211 T T T

7.3555845508880022 10.6179759444178270 20.3896485825453837 T T T

12.3706660870945537 10.6347415525452877 25.9661052106952646 T T T

10.4772224390573658 0.6754715447906924 28.5939093343994060 T T T

10.1101718043234339 1.0925935384924963 24.2882683007651252 T T T

0.5718395938252484 12.0697282017465550 9.6594860506698055 T T T

17.3066992580520207 8.2967142660372470 8.0665857491258137 T T T

4.2066392213411081 10.9710701965081956 3.4256306986389840 T T T

4.5009956305529428 0.2635025626558506 15.3435906414552612 T T T

4.3640713786204461 7.7600479049099462 4.0379463387093359 T T T

5.0043822021461102 7.9041009309002614 11.5090752953956645 T T T

0.8272608396126931 0.8609032098050693 7.1416589040375511 T T T

1.7928698253645172 12.3427671246540402 7.7039582442055030 T T T

2.1369675439802602 6.5660735654219868 8.3138688756591375 T T T

2.0329007342565353 8.2527891775719926 7.8635699133619141 T T T

7.3939962981972114 0.3483061951951705 7.9739869422668193 T T T

7.2050389858740935 0.5382321314879526 9.6880613137705645 T T T

9.2704208483678432 5.1019323450353271 8.2429650953001978 T T T

7.6339969013336049 5.7414437052391385 8.3317350367974949 T T T

3.2927978532373694 0.7248167615168697 2.1943415946885718 T T T

3.5283608079185749 0.3142092204254606 12.5158066676460429 T T T

2.7740017122961080 11.8793888994353853 1.9212728318701775 T T T

2.5786218040416924 0.0898010620710533 13.9766314808813839 T T T

3.7659916081558666 5.1821469784064602 2.6489290357132407 T T T

2.7515616737206878 5.9319387662071046 11.5941207472850945 T T T

2.9711209100000420 6.7418662799999947 2.5190865440000376 F F F

2.7335563211632485 7.6306944352760766 11.1196204076809746 T T T

5.2773429757380637 0.9279022685634402 3.8119506781158834 T T T

5.8761626227808881 0.8570733306625177 12.7577930352137656 T T T

5.7358142927483806 12.2506757936140680 4.6130424299384227 T T T

6.5910674336036301 0.7586736063410962 14.3469848853463855 T T T

5.6726570482962089 5.0791333230813835 4.1932238730302345 T T T

4.9498474899390192 5.0191573256342101 12.2843051570021675 T T T

6.1601288425423020 6.5677605881875953 4.9751524613784204 T T T

6.3101357723864435 6.1381202863392845 12.2586257531446599 T T T

9.5381117137059448 0.7143222323414004 10.0215374756295699 T T T

8.3271094516030004 7.9260408929606214 8.0764540731931156 T T T

12.8970148829080742 11.0142883799110098 3.3766711381839443 T T T

13.9139592432884971 11.9817845041046365 12.7241246357505062 T T T

13.2024695105101202 7.7641513652177672 3.9992641824076220 T T T

13.5344069480108136 8.2754108547453775 10.9915598343882461 T T T

9.6832468849664792 0.5855492924610552 7.0274499794040128 T T T

10.9780060916114479 0.7053570568406280 8.2240053511031430 T T T

11.0677726370607701 6.7519973214870035 7.8899284702534738 T T T

10.5715918141492740 8.4023262617510692 7.6675635533346185 T T T

16.5044212140397555 1.5468466754653909 8.6454183328993146 T T T

16.2785107586092614 0.5631194918017126 10.0702939052850216 T T T

0.0195933462386457 5.4026893011380706 8.7249206237288686 T T T

16.1426198254367606 6.3610690810100472 8.5031400345908796 T T T

12.0192574989919212 0.7704650063561910 2.1206880707676623 T T T

11.2773297014290890 0.3440136891654866 13.5542306267846655 T T T

11.5032232852936271 11.9268407477609308 1.8406817711907086 T T T

11.6137756906552276 11.8594656222429187 12.4183446795137282 T T T

12.5813221774145152 5.1913585002160829 2.6105937614000032 T T T

11.5615459312913380 6.0717538143268897 11.4446222632133505 T T T

11.7640209100000419 6.7418662799999947 2.5190865440000376 F F F

11.3034854922894130 7.6855192782037900 10.7895769821291374 T T T

13.9715603352715867 0.9671472504546373 3.7814337647800116 T T T

13.3121069153865577 1.6107146039047511 14.3159835331778176 T T T

14.3912961864301732 12.2898838166176692 4.6135224662638699 T T T

14.8627243723403559 0.8643280020862729 13.9856180106759940 T T T

14.5434508605185382 5.0966220454165425 4.0695379718297948 T T T

13.9132980721854054 5.5059479947098779 12.0607634127886367 T T T

15.0520375357100278 6.5854765355273361 4.8492205128391097 T T T

15.0996635786849627 6.7711424149254773 11.8737861946133343 T T T

5.0794875631126839 8.6744201910167131 26.4015811170563630 T T T

8.1915305672667866 12.2726737767538339 29.2965700833711544 T T T

10.6330986448543889 8.5834185319238880 25.9183317798356221 T T T

9.1950320419195641 9.7428017333905697 17.6487412390614509 T T T

5.2392131914586546 11.0064618642498608 20.3899165901044341 T T T

4.9998993646182122 10.6674808923654520 17.9188776301169419 T T T

9.4413126628664550 10.1691809697350362 20.1158949010086801 T T T

7.7164435988025177 12.8051859455376427 23.7568446741278123 T T T

4.9147964965205979 6.9812594489777302 23.2854089339004027 T T T

2.3455275022727720 10.8913138198150161 26.8461815896813043 T T T

10.2123433791333067 6.8956422634254730 22.8270512768336076 T T T

8.3717537678355267 9.8376430439269722 31.8619674238781734 T T T

10.6805142563812563 6.3221530838494742 28.6546799555834610 T T T

5.4267589448619411 6.4134064362921395 29.1132869683463298 T T T

4.8618232770314593 2.0164972867149453 24.3460103825216194 T T T

5.3150390768776585 1.4920817984183476 29.6131233173248312 T T T

13.4724706037118995 10.7134688320527491 25.8812987704253494 T T T

11.1425743713323246 1.3930616382974754 29.1122374029421493 T T T

10.6965365220768067 1.9080832691485106 23.8206993778247984 T T T

5.9762646579230108 10.2756924133135801 14.6033197321035093 T T T

1.0644416355775963 0.2181709775281746 7.9094392545689702 T T T

1.5900588475840176 7.4329514263707575 8.2955357449874523 T T T

7.8309278115201986 0.4794187926521250 8.8867601464807962 T T T

8.6419919101042204 5.9070946599428478 8.3541666887860355 T T T

3.3909488501393823 12.5437589887809668 2.3991751636946232 T T T

3.4993592285018336 0.1935293806709762 13.5392262170161768 T T T

3.6632391544134597 6.1467569723153153 2.9681858709595392 T T T

3.2622781003671806 6.8010427255930592 11.4018310826801486 T T T

5.0953933322552674 12.7456007222315986 3.9928728175800763 T T T

5.7483437339771752 0.7151108201201501 13.7636959412356816 T T T

5.4630550288078545 6.0554503409477709 4.4351915609498098 T T T

5.3048361405285682 5.9650188207188801 12.1396424346556344 T T T

9.9796404415462661 0.6132202412279262 8.0162719057371152 T T T

10.3414803249446585 7.4633450376432338 8.0171846627506600 T T T

16.8504830027632586 0.8003486330372729 9.2648840688157943 T T T

17.1600358073973744 6.3196602849516248 8.6306178282192842 T T T

12.1142173874100223 12.5889155566037108 2.3269700519136078 T T T

11.9645281073320646 12.5427396456958178 13.1043425840873073 T T T

12.4804547892662328 6.1533372548470258 2.9378457101240065 T T T

11.9466206883261314 6.9634102872528638 11.1251661296740973 T T T

13.7845250843374476 12.7847882256845047 3.9564488449476252 T T T

13.8414947577572587 0.8074807033212299 13.9730233770043206 T T T

14.3399482268360110 6.0708470743439014 4.3284746456799867 T T T

14.1199559822052958 6.4702264356057615 11.7918361162167376 T T T

9.3705892526338275 10.1158123756766489 30.1275454082394027 T T T

9.2765421369906473 6.8602337215813094 27.3064764821246371 T T T

5.5954138511755573 8.4175432075854300 28.9260712305549923 T T T

6.5955871837678366 6.9061523259056719 27.5412611575498012 T T T

10.5519394591545677 8.3320658884655430 28.4934501327169727 T T T

7.0997045640719021 10.1554575633588051 30.3244805360547147 T T T

8.0030929617118307 9.4086870731919419 27.6992296296625256 T T T

5.8825365997811998 9.2175121683037915 26.3803607512746474 T T T

8.1101745468036466 11.7959695084981391 28.4557642241175657 T T T

3.8980471926357012 10.8329533860761380 25.5546940648977170 T T T

6.4499015845484333 1.4903143625149244 25.4740142124862068 T T T

5.5969194701197482 12.3465754882801448 29.2184245137508931 T T T

6.6789927152178086 1.2297243306938928 28.1460657832429213 T T T

5.1749741819733206 0.0215972599742108 24.2796084119989608 T T T

4.0926621112239960 10.6076342215396178 27.8168325635785507 T T T

6.4675280362319896 11.6479281881139851 26.5701892306723089 T T T

9.8556988288343295 9.1514999456368198 26.0330258869359277 T T T

7.8608664090407885 9.3013347859971542 15.5577562209820304 T T T

6.4204471998472261 10.9189930187274076 22.5477692991208478 T T T

6.3534864446448340 7.1830869402442161 24.6860983195285151 T T T

10.1239970790354814 8.8370421948337388 23.3752251232416164 T T T

9.0410994814928998 7.1400982379269271 24.4522357729356443 T T T

5.1518629048710025 8.9163620237559584 23.8122062794401117 T T T

5.9088036903241408 10.4574488740598213 15.5851735770315969 T T T

8.6833680710748542 10.8493977381247486 22.3426107628559052 T T T

7.7518473822963792 9.6905725545453905 24.7783392034796570 T T T

11.9120474727499239 10.4771312576134310 27.1392013294277419 T T T

9.5439221500020430 1.1814112156696224 27.8955123586505209 T T T

10.3026759815459972 12.7412847753119092 23.8187019686870300 T T T

9.3215729709432225 1.4371062745361645 25.2205081846535961 T T T

10.7320432933827270 12.2594234099557315 28.7762103862918224 T T T

11.7198737703960614 10.7037756971695099 24.8763632961759029 T T T

9.3959498844659244 11.5970111728697223 26.3133381040589143 T T T

7.7706508540188661 12.1824771638070040 24.4980453666131019 T T T

6.2886594321995313 10.4425872189995914 28.2604947380800908 T T T

7.8426384048958102 8.0886121582924577 26.1068384150018531 T T T

9.8214559135249715 10.3830754326767920 27.9533832428476252 T T T

5.9894072747749103 10.7919158222284146 24.7462760961031805 T T T

7.9704196402995331 0.2707530814402109 26.5860171032151840 T T T

9.5155193350889213 10.7302258986366432 24.4343537140940228 T T T

**B4**

Sn I C H N O Zr H

1.0000000000000000

17.5857999999999990 0.0000000000000000 0.0000000000000000

0.0000000000000000 12.8148000000000000 0.0000000000000000

0.0000000000000000 0.0000000000000000 46.1650999999999954

Sn I C H N O Zr H

12 36 30 81 24 34 6 1

Selective dynamics

Cartesian

-0.0735862370279027 9.7541532177890815 3.5325448938879638 T T T

0.0882433529255827 9.2576404152386349 12.9317321583246621 T T T

-0.0844205138996604 3.5340096817463502 3.3810084763698649 T T T

0.2179799748856313 3.5177620113760781 14.0750958792597896 T T T

4.7109820806317781 10.1577188359750519 8.2695555388511988 T T T

4.7578900817053977 3.6334081122962210 9.0720394521030805 T T T

8.7194173717674754 9.7752507451577415 3.5465750688798177 T T T

9.0261878888609193 10.4571082742789443 12.8701843766980826 T T T

8.7002388409967022 3.5026549977678458 3.3824902792520870 T T T

8.8492044530390768 4.8175562100577620 15.1238869925338033 T T T

13.7830356503249298 10.4603034809965845 8.3384436716825938 T T T

13.5353105082740743 4.0324859380970546 9.0545744472926621 T T T

0.0943268820139409 7.0073784302697515 4.6197093316238620 T T T

-0.4784774092995424 6.5010637945919880 12.1377572695506935 T T T

0.4723148929255354 0.9240693460989461 4.6343628578339970 T T T

0.5749280035248585 0.7559370429256778 15.2409278372783703 T T T

5.2773095843636471 7.2693214593342166 7.7691448432136516 T T T

4.3799365288689129 0.7138563809411617 9.5614116163671792 T T T

1.4521474349999999 3.0818312520000002 0.9998610499999999 F F F

2.0691583853828335 3.0035770442159433 11.9449387497980020 T T T

1.9478911369999998 9.2808626040000011 1.4958484609999998 F F F

1.5819701483542010 9.8958760507338237 10.5335137520506770 T T T

6.8649172620327059 3.4032295312031109 7.1839960499988882 T T T

6.9939651774008906 10.9153976917073550 6.6560386982932105 T T T

5.8485974349999994 3.0818312520000002 2.4920480480000000 F F F

6.2395581128649020 3.8409029234622500 14.0816055371251796 T T T

6.3443411369999998 9.2808626040000011 1.9963422879999999 F F F

6.0957558917992074 9.9419788835299894 11.6133819114162513 T T T

2.6018945065558770 3.8394265328933814 7.1788857420799062 T T T

2.6847916163044592 10.3924023393676155 6.1981327437568732 T T T

8.9766608339640381 7.1107489785120936 4.8196426757916715 T T T

8.5359973343901139 7.0479205121283721 13.2525077191314349 T T T

9.1226329060408666 0.8116608332489698 4.4809882235764915 T T T

8.1899875610846600 0.5029142089013114 12.9571078743834178 T T T

14.1354071980546436 7.4900753812287766 8.0873954567746811 T T T

12.8887139799656509 1.2410328374723478 9.8686016109039478 T T T

10.2450474350000000 3.0818312520000002 0.9998610499999999 F F F

11.0025791952716538 3.6715300882951918 13.4342237638593414 T T T

10.7407911369999987 9.2808626040000011 1.4958484609999998 F F F

10.9643031358832985 10.6066033902076633 10.6975756613154456 T T T

15.4168403668006455 3.2534986133105739 7.0238097173832639 T T T

16.0072753411195308 10.9967435746847801 6.6340142936187876 T T T

14.6414974349999980 3.0818312520000002 2.4920480480000000 F F F

15.4630694221091272 2.7701020687401106 12.5278024130092405 T T T

15.1372411370000002 9.2808626040000011 1.9963422879999999 F F F

15.2143349090451885 10.2851303920737021 11.9248700677728667 T T T

11.2968249144311379 4.2556703402818252 7.2543886737720555 T T T

11.6865093413042587 10.6082219023327831 6.3385643083237238 T T T

0.3307327861993208 13.4467120247930811 9.2932958228795783 T T T

0.4315239489560445 7.1774206548364070 7.9581241387766379 T T T

4.1815448002396529 12.0783670367956617 3.1683365889761084 T T T

4.1335642431639661 12.8007984995440527 13.1920657963407777 T T T

4.5363307669914468 6.7090080543786303 3.7759842204182958 T T T

4.0495821353201098 6.9713020893771755 11.1709532262882885 T T T

9.2196415079720051 13.5538997176179112 9.1151103731491627 T T T

9.1656299648207344 7.5522493671187618 8.2186334854164951 T T T

13.0318904156690447 12.0625452223282803 3.1642076599293345 T T T

13.4119096721435227 12.3048749837851314 14.1666915728021809 T T T

13.2572914671142108 6.6800679113709700 3.8577385293265807 T T T

12.9867529791508254 7.0369482756612181 11.6915516795616412 T T T

10.8056629997899254 14.0773340987030018 16.8092979736615789 T T T

6.5669652576067650 13.8919623806472590 17.0914305334158847 T T T

4.4275944182681002 10.1336546534049763 18.9416003800042887 T T T

6.8415994388851296 6.7584138225116535 20.2713807380468936 T T T

6.4452591823085763 7.5055450988524219 16.1055761235234804 T T T

10.6984150338131716 10.3453743289333815 26.4433415470673587 T T T

8.1951012422245313 10.0430340415273260 25.2201260452391338 T T T

8.2712056420794369 10.2967925167947119 26.5832916636935046 T T T

10.6216331059584377 10.0932170280631812 25.0762684223663861 T T T

9.2785302252288506 9.7631926504547231 22.9827014768707087 T T T

9.5325370626881529 10.7764087237489878 28.6561170313827276 T T T

6.9069721624034743 12.9511895425319867 21.6338866080099415 T T T

11.2184283549662265 13.0697831249535508 21.2757247997646282 T T T

9.5212431832159385 10.4593762860485899 27.1999774661186571 T T T

9.3706433813550269 9.9484456039343971 24.4573914450750287 T T T

13.3935075757494548 10.4227230484900169 18.1659313426789417 T T T

11.1358488044491022 7.6745498197218867 15.7286504351008887 T T T

11.4466990675180433 6.9021066835657541 19.8945251124423663 T T T

10.4213280826762045 12.1597564997180889 14.5039093372384897 T T T

0.8888360908540036 13.3510746076287727 10.2266997844982548 T T T

-0.1977785569023945 8.0685291930019236 7.8850969810300473 T T T

4.1358659370172557 10.9948046535150432 3.2949831023268095 T T T

5.0068000148045240 12.6519612248812923 12.5517831594623370 T T T

4.3867646708551007 7.7634856933568912 4.0136752897548993 T T T

4.1362097534833122 8.0132318502137299 10.8528133301066152 T T T

0.6146105818203700 0.7394221267383801 7.2425561460214656 T T T

2.0422578031053344 13.3842782350031158 8.2522066725983869 T T T

2.3339890048023229 6.4951081103004338 7.6891488563275852 T T T

2.0716953187640792 8.2038208510208968 7.3980086085233472 T T T

7.3869919662495871 0.0723236224064679 8.4743039502127573 T T T

7.5864190382963317 13.0884446911037013 10.2053935828595002 T T T

9.1692038061013310 5.5345072445767363 8.4984389112687957 T T T

7.6001119489078119 6.3253683851352163 8.4669690561834052 T T T

3.2703295248183375 0.7819482163806966 2.0972274921268785 T T T

2.2166353488516322 -0.6189822269489371 13.5039516367870771 T T T

2.7376590881734737 11.9496123128297249 1.7897269370206808 T T T

3.0822094732080672 11.3609275509065899 12.2163787485105839 T T T

3.7949525714988885 5.1940904814518980 2.6063332306603422 T T T

2.7107432007987007 5.3920063272875129 11.2295778994102839 T T T

2.9711209099999998 6.7418662800000000 2.5190865439999999 F F F

2.1649386918807028 6.8920829836789261 10.4993692846930973 T T T

5.2322203445331521 0.9318441557612845 3.7480166987495362 T T T

3.3674637630858375 1.1200687756346239 14.6934258681769983 T T T

5.6489898223057260 12.2383352670915020 4.5463140763721022 T T T

4.9917044812138656 14.3617579204484773 14.1648864631137492 T T T

5.7407711970333803 5.1024185815410128 4.0923263167961501 T T T

5.1451700733455148 5.5044061001608107 12.1452529142892232 T T T

6.2283146382413150 6.5879438517085935 4.8904463996916121 T T T

5.9078876586614646 7.0639837333686692 11.9192138750149450 T T T

9.7933354741767449 13.7688655619213236 10.0200874717995525 T T T

8.4946477423722850 8.4043349829920313 8.0719964194352869 T T T

12.9544969027810577 10.9830884153915598 3.3072913447801509 T T T

13.1108774854769674 11.3494245003926277 14.5964754944834638 T T T

13.1228445129226312 7.7403196420377842 4.0783776808409185 T T T

13.6337480554914663 7.8234381207290733 11.2922677879978473 T T T

9.4004048412811478 0.7651391386806808 7.0442697446924258 T T T

10.8113684882607775 14.0046456393927841 7.9907251770664667 T T T

11.1091315625367901 6.9662768474520229 8.0552243502116880 T T T

10.7855374307788097 8.6451196040930967 7.7008244659736143 T T T

16.0303944647044538 0.9300623993712264 8.5473740891750563 T T T

16.1488003752090741 13.6129989220907923 10.2763988171449334 T T T

17.9294821004563509 5.1517822637558917 8.2044888767012711 T T T

16.4085350327421260 6.0284714462168054 8.3005719265481215 T T T

12.1496907387888537 0.7791468883289442 2.0894836826522734 T T T

13.0144254339274905 0.7565603661385137 12.5903251551567905 T T T

11.5535533488600617 11.9626345460386361 1.8183789793094818 T T T

12.0902473714838941 12.1069656668572083 12.6525123884320436 T T T

12.5491227847255935 5.1765865902416879 2.6536337969324602 T T T

11.0177020827134964 6.4852030282270556 11.8722489831956324 T T T

11.7640209099999993 6.7418662800000000 2.5190865439999999 F F F

11.3367669750296187 8.0022787948008318 11.0448505415665128 T T T

14.1382255146628140 0.8934809048782202 3.7029026803941343 T T T

14.8091541535367170 0.9816627519645168 14.3132198405567923 T T T

14.5319351677960267 12.1964049617947712 4.5109332364677917 T T T

14.9076698360839330 12.5235375330231982 15.5062835851266581 T T T

14.4157953514077128 5.0524632898135300 4.2424709635574933 T T T

13.0101092720814790 5.2660235686082340 12.7226434930457266 T T T

14.8877669081433233 6.5321679071010905 5.0504308722175759 T T T

14.5800057401864436 6.0256799499799012 12.4065945456062181 T T T

6.1023373842060513 12.2118853076500606 19.1599065700993947 T T T

8.6640915085555594 8.9232163647930420 15.3942071230022570 T T T

11.6312475467475238 12.3903863894873201 18.7037300771234243 T T T

11.6667982017492484 10.4680980155593115 26.9270553340367087 T T T

7.2317930083288360 9.9305062242283686 24.7250677175125624 T T T

7.3738610354431762 10.3857348565038237 27.1954339881549636 T T T

11.5232297857792449 10.0236284095469088 24.4681380233395309 T T T

9.1787181016589034 7.9191333585669010 20.9369158559891666 T T T

6.4672436023369544 13.5852449774324828 22.4286610673944864 T T T

3.3277319844748621 10.0382619179611936 19.0221191673524750 T T T

11.7454111717464116 13.7272930866298903 21.9947040924232624 T T T

11.2012729312738681 14.9924278070344315 16.3263949071606724 T T T

6.0749815613543072 14.7753570255878373 16.6384562647595260 T T T

6.2928412045507969 5.8766494335216608 20.6551651539152665 T T T

5.8221125046138296 6.7873344744619697 15.5394518476482180 T T T

14.4977204748073962 10.3754440359416478 18.0758782632402948 T T T

11.7321210919742782 6.9973923425271476 15.0856131914437093 T T T

12.1060065132419457 6.0594545420293935 20.1816106831353608 T T T

6.2724202451444775 10.3469435743693108 14.0837069156975083 T T T

6.7842148097587183 11.8216856322447157 14.4023258950848803 T T T

1.0229332653418730 13.4456568597115460 8.1842658145867375 T T T

1.7298944697303735 7.3184307141962925 7.7973851935831275 T T T

7.9694442401835737 13.1767970851657239 9.2656979034213194 T T T

8.6203057324863490 6.3988366710425524 8.5473418904577478 T T T

3.3620371527863000 12.5956891144280334 2.2795070477459531 T T T

3.0889714417710725 12.0290334565540959 12.9925801517247947 T T T

3.6854937433118256 6.1532750225949782 2.9394686338816793 T T T

2.9130165197330755 6.3576140407419084 10.9367542573328400 T T T

5.0412988380517110 12.7473985075250287 3.9034183893666792 T T T

4.1902195128414510 13.7254626338241117 14.1228146474132608 T T T

5.5298708319010466 6.0746521760190308 4.3506257584639183 T T T

5.0943250472618038 6.4587051893546477 11.7799870673671450 T T T

9.8378381623938331 13.7005385475663157 7.9709856957045995 T T T

10.4606423348769120 7.7581349589679789 8.1117763886755672 T T T

16.6058265935496259 13.5555180027458348 9.3709672579762628 T T T

17.4295648895629434 6.0428428580501006 8.2726886712273284 T T T

12.2077123028410508 12.5937988304968531 2.2877035154604437 T T T

12.7462214166240990 12.7382432029886878 13.1234746677718181 T T T

12.4446982505427197 6.1401905773965284 2.9764298418757229 T T T

11.6932993147473709 7.1439995984060687 11.4796291813514717 T T T

13.9297175275283962 12.7135926876444376 3.8692741261739019 T T T

14.3977223967292183 12.9593035858975014 14.7472490369887996 T T T

14.1989188133930355 6.0243530110924883 4.4964204536100807 T T T

13.5558250320503433 6.0547480801968421 12.3532852504933928 T T T

10.4371140344185278 11.1838879320384326 14.5960545673489630 T T T

10.0570973698348336 14.2443833437724425 17.8216175097567167 T T T

6.2323903856732503 12.7563378103606109 16.6285224012985928 T T T

7.4050432784538067 14.1325699204603339 18.0146852496587861 T T T

11.1522469348655431 12.9718891465710087 16.2856809805907190 T T T

6.5447454245100598 10.9544163068043954 14.8248259493576970 T T T

8.7444378512729699 11.6825687181575706 17.2729469779876617 T T T

6.8946114730829233 11.6748747203804371 19.0014852326014783 T T T

8.7289653479394431 9.4342622683052575 16.2291624061015121 T T T

5.0881475679504291 9.9512178790139263 20.0088883162746960 T T T

7.6591715993385847 6.5421311360139773 19.3239861153399666 T T T

6.0891405118500188 8.7165559552652816 16.0938561500686639 T T T

7.4622652736358042 7.0082192496681754 16.7025308510107529 T T T

6.5836823563811864 7.8674340191779866 20.8369724220800521 T T T

4.8808242062936076 10.4237966938264552 17.7886090995559769 T T T

7.4564306773806228 9.2843412009052528 18.4862423015531618 T T T

10.8604209511503118 11.8030885404839623 18.6646747938037940 T T T

10.7916745413464863 10.9266651857950912 29.1606086101958084 T T T

8.1045248875374440 9.6604244507189296 22.4780820338995753 T T T

7.6435312857936655 13.5442462361472629 20.7858110438111581 T T T

11.6005169440798586 11.8574575346516671 21.2432208917914451 T T T

10.3118181437372609 13.6162712806426427 20.5753898797454617 T T T

6.6076498956814076 11.7167848564748311 21.6725210749556894 T T T

8.5294083008603803 10.9072239425622577 29.3408962181592621 T T T

10.3639230224995504 9.7413935352112500 22.3055811821382726 T T T

9.0121245206008265 11.0866834006674821 20.1904538249550320 T T T

12.7587506924406053 10.7191000051415184 17.1009315150197061 T T T

10.2546834801504154 7.1061985624723496 16.4641720423021489 T T T

11.7185025481363994 8.0294918330433713 20.4150669548482533 T T T

10.5039567088659087 6.6258087860322403 19.0891225920279304 T T T

11.3911213039802544 8.9053950970818612 15.6558292794952720 T T T

12.9179595357422681 10.1858116111130510 19.3150266510256898 T T T

10.3835868380433496 9.3718772506839727 18.2326432474650453 T T T

9.0994706936036813 8.5941190233106060 20.2448541149067864 T T T

6.9547558327731913 10.6277633258541186 17.0123280005215030 T T T

8.8652350899215886 12.8156867519784381 19.0525512116147269 T T T

10.5342243871404904 10.7751687917530798 16.6782339126601471 T T T

7.2883320783151513 9.9573518849626765 20.4611883863831210 T T T

8.9515203833296777 7.9075308193737417 18.0987254649286875 T T T

10.8204666962159539 10.0660593852017399 20.1497059377409720 T T T

10.6752067359130614 11.1425123882595205 30.1097264451640179 T T T
